# Modulating Thin Film Transistor Characteristics by Texturing the Gate Metal

**DOI:** 10.1038/s41598-017-18111-5

**Published:** 2017-12-20

**Authors:** Aswathi Nair, Prasenjit Bhattacharya, Sanjiv Sambandan

**Affiliations:** 1 Department of Instrumentation and Applied Physics, Indian Institute of Science, Bangalore, 560012 India; 2 Department of Engineering, University of Cambridge, Cambridge, CB3 0FF United Kingdom

## Abstract

The development of reliable, high performance integrated circuits based on thin film transistors (TFTs) is of interest for the development of flexible electronic circuits. In this work we illustrate the modulation of TFT transconductance via the texturing of the gate metal created by the addition of a conductive pattern on top of a planar gate. Texturing results in the semiconductor-insulator interface acquiring a non-planar geometry with local variations in the radius of curvature. This influences various TFT parameters such as the subthreshold slope, gate voltage at the onset of conduction, contact resistance and gate capacitance. Specific studies are performed on textures based on periodic striations oriented along different directions. Textured TFTs showed upto ±40% variation in transconductance depending on the texture orientation as compared to conventional planar gate TFTs. Analytical models are developed and compared with experiments. Gain boosting in common source amplifiers based on textured TFTs as compared to conventional TFTs is demonstrated.

## Introduction

Thin film transistors (TFTs) based on disordered semiconductors are the building blocks of integrated circuits on large area flexible substrates. Advances in materials and fabrication methods have resulted in expanding the application spectrum from the traditional applications such as displays^1–3^ and image sensors^4–6^ to applications in data transmission and storage systems^7–14^ wearable electronics such as smart bandages and other health care monitoring devices^15–20^ radio frequency identification^21–23^ wearable energy harvesting systems^24–27^ sensors and actuators on elastomers^28–30^ etc. To enable these applications, the design of reliable TFT circuits have become important.

An important consideration for good circuit design is the ability to control the transconductance of the TFT. A simple, passive means of controlling TFT transconductance is the control of the TFT aspect ratio (gate bias control being more active). Aspect ratio scaling however requires the use of increased layout area which reduces the spatial resolution. This is particularly important if the circuit is a part of the pixel circuit in an active matrix architecture. Moreover, increasing channel width can increase the parasitic overlap capacitance. Consider the example of a common source voltage amplifier using a non-complementary driver and load. To achieve a small signal gain of *G* > 1 the layout area of the circuit will have to scale by a factor of 2*G* (increasing channel width of the driver and channel length of the load simultaneously) thereby reducing spatial resolution. This would also increase the gate-drain overlap capacitance of the drive TFT by a factor of *G* and therefore increase the input Miller capacitance by a factor of *G* ^2^ resulting in a stronger pole at the input. Therefore, although the control of aspect ratio is most practical and convenient, we consider other means to control the transconductance of the TFT and also improve TFT performance without changing the semiconductor material.

To engineer TFTs and TFT circuits with improved control and performance, several techniques have been proposed. These approaches can be classified into four types. First are those techniques that modify the source drain contacts. For example, Shannon *et al*. proposed a modified TFT structure with a reverse biased source electrode to achieve high performance devices using low mobility semiconductors^31^. By creating Schottky barrier contacts and operating in deep sub-threshold, Lee *et al*. developed TFTs with high transconductance and TFT amplifiers with high output impedance resulting in high gain amplifier circuits^32^. The second class of techniques consists of device and circuit engineering designed to overcome the handicap of the absence of a complementary TFT. For example, a full swing inverter without employing complementary technology was realized using an enhancement type driver and a depletion type load by Han *et al*.^33^. Sambandan engineered high gain amplifiers using n-type TFTs by the use of positive feedback to develop a pseudo p-type TFT based current source^34^. High gain operational amplifiers were also realized by incorporating a similar positive feedback technique^35–37^. Shoute *et al*. implemented TFTs that behaved similar to conventional field effect transistors by sustaining a strong p-type inversion layer in n-type metal oxide TFTs^38^. Cantatore *et al*. and Huang *et al*. suggested design rules for implementing logic gates without complementary TFT technology^39,40^. Munzenrieder *et al*. realized a fully integrated static random access memory using only n-type TFTs^41^. The third approach consists of a miscellany of fabrication techniques or operation methods or a combination of both. For example, Seo *et al*. realized a ring oscillator at 165 MHz using TFTs with a nanotrench structure fabricated using nano imprint lithography resulting in a three dimensional TFT architecture with low channel length^42^. By operating in a non-quasi-static mode, Rotzoll *et al*. developed a full wave rectifier circuit with organic TFTs operating at 13.56 MHz^43^. Cai *et al*. developed TFTs with a highly polarizable insulator offering high gate capacitance leading to a high on-off ratio^44^. The fourth class of techniques, that is also of interest to this work, considers the out-of-plane patterning of the geometry of the metal-insulator-semiconductor stack to modulate TFT performance. Sambandan found that creating periodic corrugations on the gate metal and thereby influencing the metal-insulator-semiconductor stack influenced the performance of TFTs^45^. Aljada *et al*. also realized the same phenomena by patterning the metal-insulator-semiconductor stack with dimples^46^. Rex *et al*. found that such patterning could either enhance or degrade TFT performance depending on the geometry which in turn influenced the effective insulator capacitance^47^. Studies on TFTs developed on paper by Martin *et al*. provided further evidence that roughness induced deformations of the metal-insulator-semiconductor stack tended to improve TFT performance due to improvements in the gate capacitance^48^. Sekine *et al*. studied the TFT behavior under a strain induced deformation of the TFT stack and found that the strain contributed to an increase in contact resistance of the device^49^.

In this work we investigate the modulation of TFT transconductance by the purely geometrical approach of texturing of the gate metal resulting in non-planar metal-insulator-semiconductor stacks. This interaction between geometry and functionality has strong applications. Reconsider our example of the common source amplifier. By texturing the gate of the driver and load TFTs differently, it is possible to obtain the desired gain of *G* > 1 by no or minimal variations in the aspect ratios of the TFTs. For modest gains, it is entirely possible to maintain the channel width and channel length of all TFTs at the minimum feature size and still obtain *G* > 1.

From the point of view of fabrication, the texturing of the metal-insulator-semiconductor stack is achieved by a dual gate metal deposition. The first gate metal deposition results in a planar layer. The second gate metal deposition occurs over the first planar layer and is patterned to achieve the desired texturing. Although this work illustrates this concept using conventional amorphous hydrogenated silicon (a-Si:H) TFTs, the fabrication of textured TFT is much easier to achieve with techniques such as ink-jet printing or stamping.

The major contributions of this work are as follows. Firstly, we study the impact of gate texturing based on periodic striations oriented along different directions with respect to the channel length. The variations of TFT parameters on the texture directionality is investigated analytically and experimentally. Second, detailed analytical models for the modulation of surface potential and free carrier concentration due to texturing are presented via the solution of the Poisson-Boltzmann equation in polar co-ordinates. These results are finally used to develop a model for the current-voltage characteristics. Furthermore, intuitive semi-empirical geometric models are also defined to quickly determine TFT parameters due to texturing. All models are corroborated with experiments. Finally, the concept of texture based gain control in TFT voltage amplifiers is demonstrated.

## Results and Discussions

### Device Geometries

**Figure 1:**
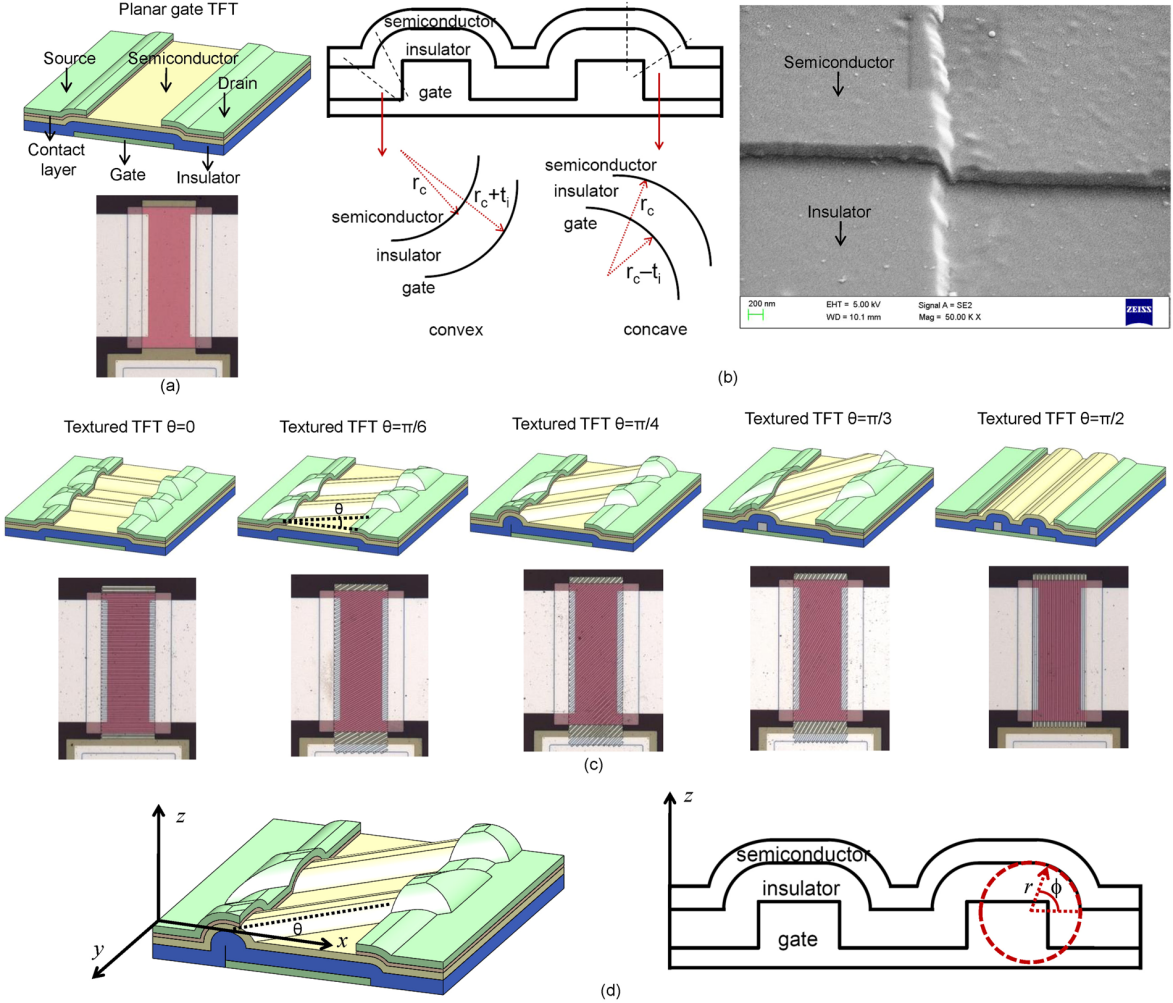
(**a**) Schematic diagram and micrograph of TFT with planar gate. (**b**) Schematic of the cross-section of a textured gate TFT. The local radius of curvature, *r*_*c*_(*x*, *y*), is defined at the semiconductor-insulator interface for all regions with *r*_*c*_ being infinite in the planar regions. A scanning electron microscopy image for the metal-insulator-semiconductor stack for a fabricated textured TFT is also shown. (**c**) Schematic and micrographs of textured TFT with striated texturing at *θ* = 0, *θ* = *π*/6, *θ* = *π*/4, *θ* = *π*/3 and *θ* = *π*/2. (**d**) Co-ordinate systems used in the analysis.

Figure 1 illustrates the geometries of planar (Fig. 1a) and textured gate (Fig. 1b and c) a-Si:H TFTs that were fabricated using the process flow as described in the Methods section. The texturing was primarily designed in the form of periodic striations oriented along different directions. The schematic cross-section of metal-insulator-semiconductor stack after texturing is shown in Fig. 1b. The metal-insulator-stack in the fabricated devices was conformal as shown in the SEM image with the silicon nitride insulator having a thickness *t*_*i*_ = 200 nm. The SEM images of multiple devices are given as Fig. S1 in the Supporting Information. There exist three types of interfaces. Interfaces where the semiconductor presents a convex face to the insulator (convex regions), interfaces of zero curvature where the metal-insulator-semiconductor stack is planar (planar regions, also as expected in a conventional planar gate TFT) and interfaces where the semiconductor presents a concave face (concave regions) to the insulator. We define the insulator-semiconductor interface as having a local radius of curvature *r*_*c*_(*x*, *y*). For the textured a-Si:H TFTs fabricated for experiments in this work, the typical minimum value of *r*_*c*_(*x*, *y*) ~ 250 nm. Defining the *x*-direction and *y*-directions to be along the channel length and along the channel width, respectively, the striations were made to lie along an angle *θ* that an in-plane vector makes to the *x*-direction with 0 ≤ *θ *≤ *π*/2 as shown in Fig. 1c. The striations were also made to extend below the source drain contacts. All TFTs used in this study had a channel width of *W* = 400 *μ*m. The striations had a height of 200 nm, planar width of 4 *μ*m and a pitch of ~12 *μ*m. Figure 1d shows the coordinate systems used in this work. For the convex and concave regions we also a define a polar co-ordinate system (*r*, *ϕ*) with radial coordinate *r* as it greatly simplifies the analysis. The polar coordinate system maps with the Cartesian system as *x* = *r*cos(*ϕ*)sin(*θ*), *y* = *r*cos(*ϕ*)cos(*θ*) and *z* = *r*sin(*ϕ*).

### Experimental Results

**Figure 2:**
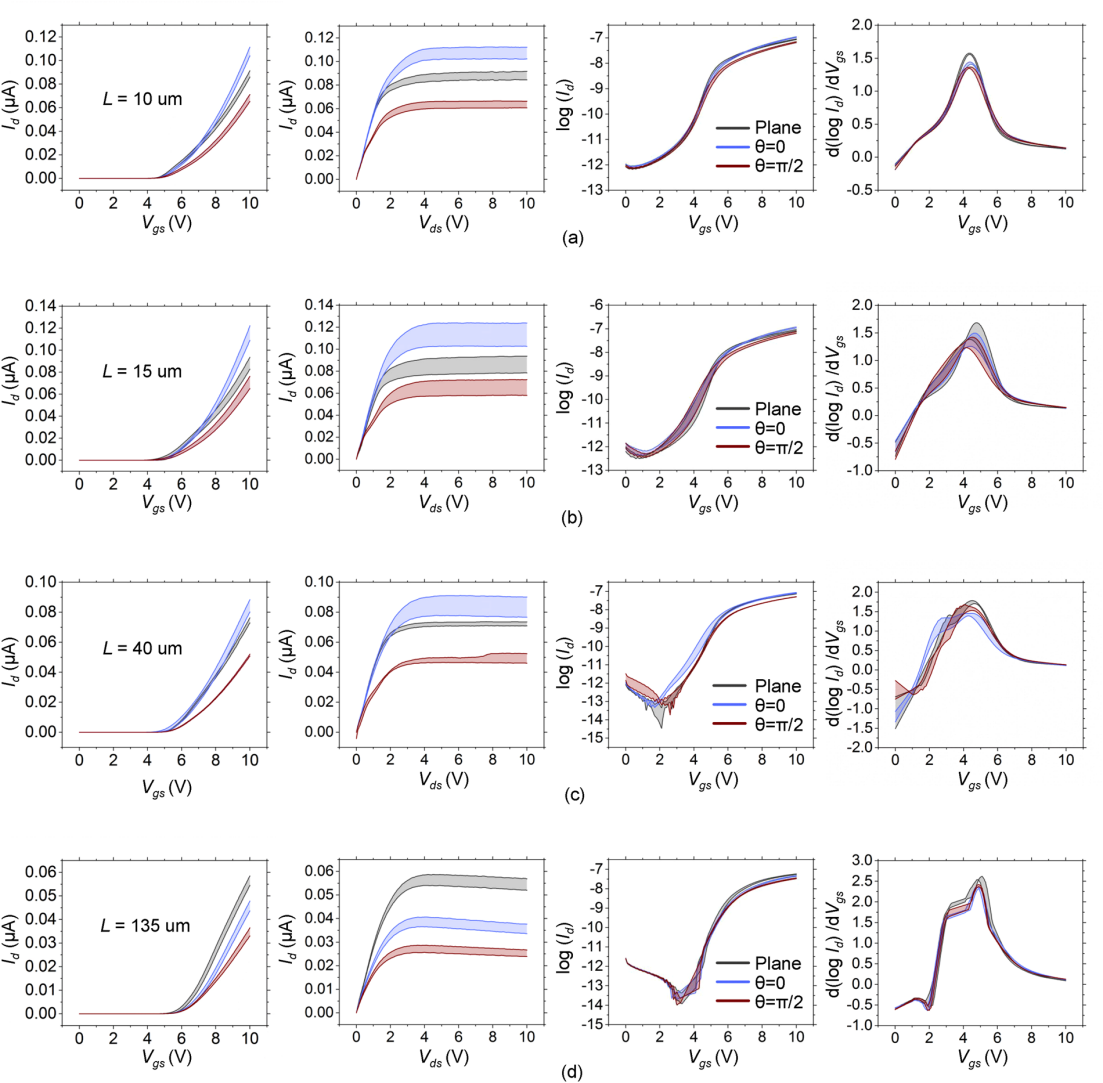
Error band plots for transfer characteristics at *V*_*ds*_ = 2 *V*, output characteristics at *V*_*gs*_ = 10 *V*, transfer characteristics at *V*_*ds*_ = 2 *V* in log scale, derivative of transfer characteristics in log scale of plane, *θ* = 0 and *θ* = *π*/2 striated devices for different channel lengths (**a**) *L* = 10 *μm*. (**b**) *L* = 15 *μm*. (**c**) *L* = 40 *μm*. (**d**) *L* = 135 *μm*.

Figure 2 shows the current-voltage characteristics (I–V characteristics) of TFTs with planar and textured gates with texturing along *θ* = 0 and *θ* = *π*/2. The characteristics are shown for TFTs of different channel lengths, *L* = 10 *μ*m (Fig. 2a), *L* = 15 *μ*m (Fig. 2b), *L* = 40 *μ*m (Fig. 2c) and *L* = 135 *μ*m (Fig. 2d). Each plot shows the band of standard error obtained by testing four devices for each case and provides a reasonably good estimate of the typical performance of the device. The color codes of grey, blue and red are used to indicate the characteristics of planar gate TFTs, textured gate TFTs with striations along *θ* = 0 and textured gate TFTs with striations along *θ* = *π*/2, respectively. The labels *I*_*d*_, *V*_*gs*_ and *V*_*ds*_ indicate the drain-source current, gate-source voltage and the drain-source voltage respectively. Figure 2 shows the transfer characteristics in linear scale obtained at *V*_*ds*_ = 2 V, output characteristics obtained at *V*_*gs*_ = 10 V, transfer characteristics in log scale, and the plot of dlog*I*_*d*_/d*V*_*gs*_ versus *V*_*gs*_ to help identify the sub-threshold slope (peak of the curves), onset of sub-threshold and above threshold conduction (threshold voltage). The mobility for planar TFTs was observed to be about 0.19 cm^2^/Vs. Detailed data sets are presented as Figs S2–S5 in the Supporting Information.

**Figure 3:**
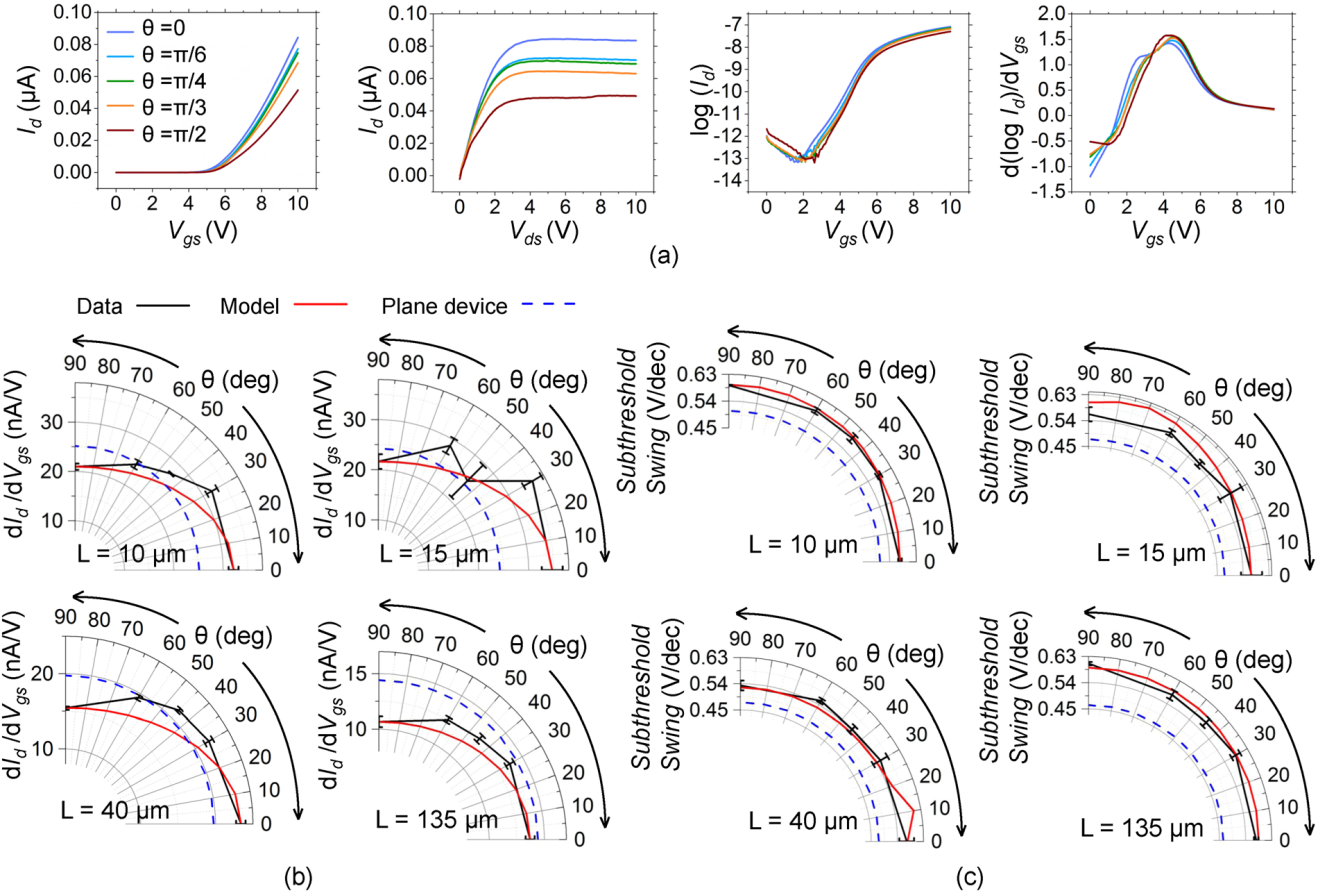
(**a**) I–V characteristics of textured TFTs with striated texturing along different *θ*. Transfer characteristics on a linear scale measured at *V*_*ds*_ = 2 V, output characteristics measured at *V*_*gs*_ = 10 V, transfer characteristics on a log scale and the plot of d*logI*_*d*_/d*V*_*gs*_ versus *V*_*gs*_ for channel length of 40 *μ*m. Impact of *θ* on (**b**) Effective transconductance (**c**) Sub-threshold slope. Parameters extracted from experiment for textured TFTs (black solid line with markers), elliptical model (red solid line), corresponding parameter measured in planar gate TFT (blue dashed line).

Figure 3 shows the impact of *θ* on the performance of textured TFTs. Using textured TFTs of *L* = 40 *μ*m channel length having texturing with different *θ*, Fig. 3a shows the transfer characteristics on a linear scale measured at *V*_*ds*_ = 2 V, output characteristics measured at *V*_*gs*_ = 10 V, transfer characteristics on a log scale and the plot of dlog*I*_*d*_/d*V*_*gs*_ versus *V*_*gs*_. Figure 3b and c define the variation of the effective transconductance d*I*_*d*_/d*V*_*gs*_ and sub-threshold swing, respectively. These parameters are extracted from the I–V characteristics and are shown in black markers with error bars for *θ* of 0, *π*/6, *π*/4, *π*/3 and *π*/2. The red solid curve in these plots indicate the fit of the elliptical model. The blue dashed curve indicates the value of the parameter for the planar gate TFT. The methods of extraction of these parameters as well as variations of threshold voltage and leakage current are discussed in the Supporting Information.

It is observed from Fig. 2 that TFTs with texturing along *θ* = 0 show higher currents as compared with texturing along *θ* = *π*/2 for all channel lengths. The above threshold current of textured TFTs with texturing along *θ* = 0 is higher than planar gate TFTs for small channel lengths but lower than planar gate TFTs for large channel lengths. This trend is reflected in the plots of the transconductance for various *θ* as shown in Fig. 3. Furthermore, a trend is observed with the effective transconductance decreasing with increasing *θ*. It is also seen in both Figs 2 and 3 that the subthreshold swing is typically lower (~0.45 V/dec) in the conventional planar gate TFTs as compared to textured gate TFTs. Variations in *θ* do not appear to affect the subthreshold swing significantly and the value remains at ~0.54 V/dec for all *θ*. The threshold voltage too appears unaffected significantly by texturing and is similar (~4.5 V) for both planar and textured TFTs. It is also unaffected by variations in *θ* (Figure S6 Supplementary Information). The textured TFTs show a significantly larger drain-source leakage current as compared to planar gate TFTs. However, the variations in observed leakage currents are too large to discern any trends with variations in *θ* (Figure S6 Supplementary Information).

### Spatial Modulation of Parameters due to Texturing

#### Modulation of Local Gate Capacitance per Unit Area, *c*_*i*_(*x*, *y*)

First we consider the impact of texturing on capacitance of the metal-insulator-semiconductor stack. Since texturing results in the semiconductor-insulator interface having convex, concave and planar regions, i.e. having variations in the local radius of curvature *r*_*c*_(*x*, *y*), the capacitance per unit area also becomes a function of *x* and *y*. We define the local value of this capacitance per unit area at any point (*x*, *y*) and for the elemental section d*x* by d*y* by the variable *c*_*i*_(*x*, *y*). The total capacitance of the elemental section is therefore *c*_*i*_d*x*d*y*.

In regions where the metal-insulator-semiconductor stack remains planar (as also in the case of conventional planar gate TFTs), *c*_*i*_(*x*, *y*) = *ε*_*i*_/*t*_*i*_ with *ε*_*i*_ being the permittivity of the insulator and *t*_*i*_ the thickness. In regions where the semiconductor-insulator interface is convex and having a local radius of curvature, *r*_*c*_, the metal-insulator-semiconductor stack can be modeled as a cylindrical capacitor *c*_*i*_(*x*, *y*) ~ *ε*_*i*_/(*r*_*c*_ln(1 + (*t*_*i*_/*r*_*c*_))). In regions where the semiconductor-insulator interface is concave with a local radius of curvature, *r*_*c*_, *c*_*i*_(*x*, *y*) ~ *ε*_*i*_/(−*r*_*c*_ln(1−(*t*_*i*_/*r*_*c*_))). In general it can be shown that *c*_*i*_ in the convex region is greater than *ε*_*i*_/*t*_*i*_ which in turn is greater than the value of *c*_*i*_ in the concave region. When *r*_*c*_ ≫ *t*_*i*_, *c*_*i*_ ~ *ε*_*i*_/*t*_*i*_ for all regions.

For striated texturing along *θ*, *r*_*c*_ varies along the *π*/2 − *θ* direction while remaining infinite along the *θ* direction and *c*_*i*_ is also expected to vary along the *π*/2 − *θ* direction. This spatial modulation in the local gate capacitance per unit area is responsible for a range of observations.

#### Modulation of Surface Potential, *φ*_*s*_

The density of gap states in the semiconductor of the TFT can be described with an exponential distribution in energy having a characteristic temperature and an equivalent characteristic voltage, *V*_*tc*_. The local potential profile, *φ*, in the semiconductor would be dependent on the geometry of the metal-insulator-semiconductor stack and is expected to vary with *r*_*c*_. For a given *φ*, the total carrier concentration per unit volume trapped in the states swept by the Fermi level can be written as ${n}_{t}={n}_{t0}{e}^{\phi /{V}_{tc}}$ with *n*_*t*0_ defining the trapped carrier concentration at flat-band. The free carrier concentration can be defined as ${n}_{f}={n}_{f0}{e}^{\phi /{V}_{th}}$ where *n*_*f*__0_ is the free carrier concentration at flat-band with *V*_*th*_ < *V*_*tc*_ being the thermal voltage. The electrostatics of the device is defined by a Poisson-Boltzmann like equation ∇^2^ *φ* = *q*(*n*_*t*_ + *n*_*f*_)/*ε*_*s*_ with *ε*_*s*_ being the permittivity of the semiconductor. In the sub-threshold operation, the Fermi level is located closer to mid gap with *n*_*f*_ ≪ *n*_*t*_. However, since *V*_*th*_ < *V*_*tc*_, a large enough gate voltage could result in *n*_*f*_ ≫ *n*_*t*_^50^. In this analysis, we assume that the electrostatics of the TFT during turn on and just after turn on is mostly dictated by the carriers trapped in the gap states. On the other hand, the I–V characteristics of the device is defined by *n*_*f*_ due to the higher mobility of free carriers.

The solution of Poisson’s equation for the case of a planar metal-insulator-semiconductor stack is well known^51^. For the convex and concave regions we use a polar co-ordinate system (*r*, *ϕ*) with radial coordinate *r* as shown in Fig. 1d. The Poisson Boltzmann equation defining the electrostatics can now be written in polar form as,

1 $${d}^{2}\phi /d{r}^{2}+\mathrm{(1/}r)(d\phi /dr)=q({n}_{t}+{n}_{f})/{\varepsilon }_{s}$$

It can be shown that (See Supporting Information), *φ*(*r*) ~ 2*V*_*tc*_ln((*κl*_*tc*_/*r*)sec(*κ*ln(*r*/*l*_*b*_))). Here *l*_*b*_ is a characteristic length that scales with *r*_*c*_ with $\,\mathrm{lim}\,{}_{{r}_{c}\to \infty }({l}_{b}/{r}_{c})=1$ and *l*_*tc*_ = (2*ε*_*s*_ *V*_*tc*_/*qn*_*t*0_)^1/2^ is a characteristic length equivalent to a Debye length. Also, *κ* ^2^ = ((*r*_*c*_ ± *t*_*s*_)/*l*_*tc*_)^2^ − 1 where + is for the concave case, − is for the convex case and *t*_*s*_ is the thickness of the semiconductor. The boundary condition is given by Gauss’ law to be *c*_*i*_(*V*_*gs*_ − *V*_*fb*_ − *φ*(*r* = *r*_*c*_)) = ±*ε*_*s*_ *ξ*(*r* = *r*_*c*_) with *V*_*gs*_ is the applied gate-source bias, *V*_*fb*_ the flat-band voltage and *ξ* = −∇*φ* the electric field in the semiconductor that is a function of *φ* and the channel potential. To include the effect of the channel potential, *V*_*ch*_, all potentials can be referenced to the source electrode. Subsequent to the application of this boundary condition, the surface potential at any point in the channel, *φ*_*s*_, is described by the Lambert-W function, **W**_**0**_, as

2 $${\phi }_{s}={V}_{gs}-{V}_{fb}-{V}_{ch}-2{V}_{tc}{{\bf{W}}}_{{\bf{0}}}(\frac{{\varepsilon }_{s}}{{l}_{tc}{c}_{i}(x,y)}{e}^{({V}_{gs}-{V}_{fb}-{V}_{ch})/2{V}_{tc}})$$

when *V*_*gs*_ − *V*_*fb*_ − *V*_*ch*_ ≫ 0, we can approximate **W**_**0**_(.) ~ ln(.) − ln(ln(.)) and the surface potential is found to scale as,

3 $${\phi }_{s}\sim 2{V}_{tc}\,\mathrm{ln}(\frac{{c}_{i}({V}_{gs}-{V}_{fb}-{V}_{ch})}{q{n}_{t0}{l}_{tc}})$$

**Figure 4:**
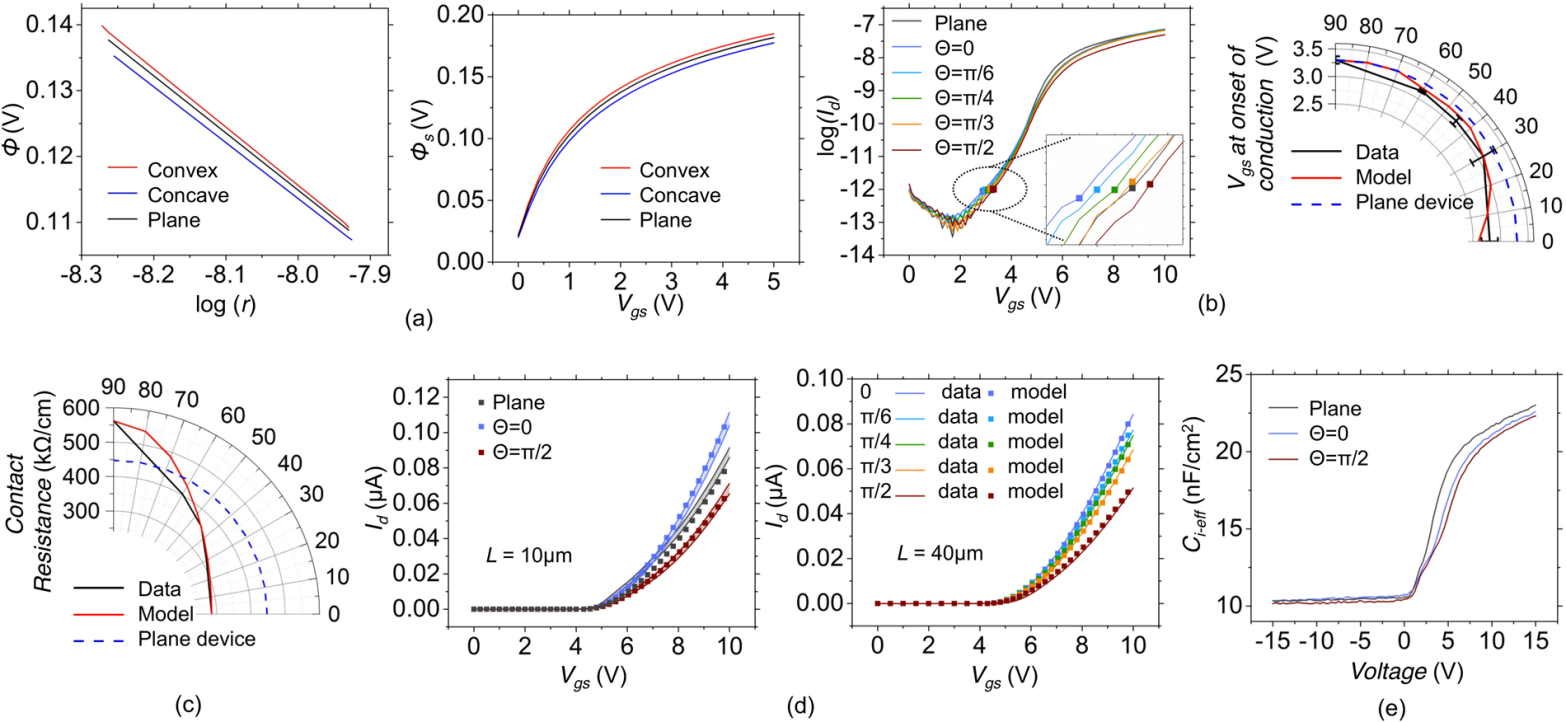
(**a**) TCAD simulations illustrating the modulation of the potential for the convex, planar and concave regions as a function of the polar co-ordinate *r* (normal to the gate metal interface). Also shown are simulation results defining the variation of the surface potential with *V*_*gs*_ for the convex, planar and concave regions. (**b**) Experimentally determined variations of the *V*_*gs*_ at the onset of sub-threshold conduction in textured TFTs with *L* = 40 *μ*m as a function of *θ*. Textured TFTs with striations oriented along *θ* = 0 turn on the fastest while those with striations along *θ* = *π*/2 turn on the slowest. (**c**) Impact of texturing on the contact resistance. (**d**) Comparison of the experimentally determined I–V characteristics with the analytical model (markers). (**e**) Experiments determining the cumulative impact of texturing on the C-V characteristics.

In summary, the spatial modulation of the surface potential is due to the spatial modulation of *c*_*i*_. If however, *V*_*gs*_ − *V*_*fb*_ − *V*_*ch*_ ~ 0, **W**_**0**_(0) ~ 0, and *φ*_*s*_ ~ *V*_*gs*_ − *V*_*fb*_ − *V*_*ch*_. Figure 4a shows ATLAS TCAD simulations of the expected variations in surface potential in the convex, planar and concave regions.

#### Modulation of Free Carrier Concentration per Unit Area, *N*_*f*_

The discussions so far has looked at the case of the TFT during turn on with an implicit assumption that *n*_*t*_ + *n*_*f*_ ~ *n*_*t*_. However for large *V*_*gs*_, the Fermi level would have swept through the gap states and moved significantly close to the mobility edge and it is possible that *n*_*f*_ ≫ *n*_*t*_. Therefore the charge on the gate is compensated for by both, the free carriers and the trapped charge. If the assumption *n*_*t*_ + *n*_*f*_ ~ *n*_*f*_ is made, one must account for all the trapped charge. This is done by defining a threshold voltage, *V*_*fb*_ + *qN*_*t*_/*c*_*i*_, with *N*_*t*_ being the trapped carrier concentration per unit area and appropriately modifying *φ*_*s*_. The free carrier concentration per unit area, ${N}_{f}=-{\int }_{{\phi }_{s}}^{0}({n}_{f}/\xi ){\rm{d}}\phi$, can now be defined for both cases, *n*_*t*_ ≫ *n*_*f*_ and *n*_*f*_ ≫ *n*_*t*_, in a generic manner by the use of the variable *V*_*on*_ as,

4 $$q{N}_{f}=\gamma {({c}_{i}({V}_{gs}-{V}_{on}-{V}_{ch}))}^{\alpha }$$

when *n*_*t*_ ≫ *n*_*f*_ (eg. in sub-threshold operation), *γ* = (1/*α*)(*n*_*f*0_/*n*_*t*0_)(*qn*_*t*0_ *l*_*tc*_)^1−*α*^, *α* = (2*V*_*tc*_/*V*_*th*_) − 1 and *V*_*on*_ = *V*_*fb*_. On the other hand, when *n*_*f*_ ≫ *n*_*t*_, *γ* = 1, *α* = 1 and *V*_*on*_ = *V*_*fb*_ + *qN*_*t*_/*c*_*i*_, i.e. a local threshold voltage. Better estimates of *γ*, *α* and *V*_*on*_ are obtained by smoothing the two regions. Once again it is observed that the spatial modulation of *c*_*i*_ not only results in the spatial modulation of the surface potential but also the free carrier concentration.

#### Modulation of the Field Effect Mobility, *μ*

In crystalline semiconductor based field effect transistors, the field effect mobility degrades with an increase in gate voltage due to the increased interaction of carriers with the insulator^52^. However, the mobility of carriers in disordered semiconductors is defined by the multiple-trap-release mechanism and it is well established that the mobility, *μ*, increases with increased field effect^53^. It is only at significantly high electric fields that any mobility degradation is observed with insulator trapping being a contributor^54,55^. This increase in field effect can be realized by either increasing the gate voltage or by increasing *c*_*i*_. Since texturing modulates *c*_*i*_, it is expected that the carriers present in the convex regions experience higher field effect mobility as compared to the planar regions. The opposite is true for the carriers in the concave regions. The impact of texturing on *μ* can be quantified by the relation *μ* = *μ*_0_(*c*_*i*_ *t*_*i*_/*ε*_*i*_)^*ν*^, with *μ*_0_ being the field effect mobility observed in the planar regions, and *ν* being a constant coefficient. Once again, the spatial modulation of *c*_*i*_ results in the spatial modulation in *μ*.

### Cumulative Impact of Texturing: I–V Characteristics

The previous section discussed the spatial modulation of TFT parameters due to texturing. It was established that the variations in *r*_*c*_ in *x* and *y* directions resulted in modulating *c*_*i*_, *φ*_*s*_, *N*_*f*_ and *μ* in *x* and *y* directions. However, it is the integral or cumulative effect of these spatial modulations that determine the overall performance of the TFTs. In this section we define the cumulative effect of the local modulations of these parameters and derive the I–V characteristics. Subsequently, attempts are made to explain the trends observed in Figs 2 and 3.

#### Impact on Effective Channel Width and Channel Length

The modulation of the semiconductor-insulator interface out of plane implies that the effective channel width and effective channel length are now defined by the path length of the path traversed along the semiconductor-insulator interface in the *y*-direction and *x*-direction, respectively. We define this effective channel width and channel length as *W*_*θ*_ and *L*_*θ*_, respectively, with the subscript *θ* used to define the orientation angle of the striation and takes the appropriate value for the particular case. If the texturing were removed, both *W*_*θ*_ and *L*_*θ*_ would equal the plane projected channel width *W* and *L* of a planar gate TFT, respectively. For periodic striated texturing, let *s* be the projected spatial width of one period in the *π*/2 − *θ*-direction, and *s*_*θ*_ the path length while traversing a path along the semiconductor-insulator interface in the *π*/2 − *θ* direction. Then, *W*_*θ*_ = *s*_*θ*_ *W*/*s* for all *θ* < *π*/2. For *θ* = *π*/2, *W*_*θ*_ = *W*_*π*/2_ = *W*. Also, *L*_*θ*_ = *s*_*θ*_ *L*/*s* for all *θ* > 0. For *θ* = 0, *L*_*θ*_ = *L*_0_ = *L*. In the particular case of the devices fabricated in this work, *s*_*θ*_ = 12.6 *μ*m and *s* = 12 *μ*m. Compared to planar gate TFTs, this results in a 5% improvement and attenuation of performance for textured TFTs for *θ* = 0 and *θ* = *π*/2, respectively.

#### Impact on the Onset of Conduction

Texturing results in the convex regions having a higher values for *N*_*f*_ and *μ* as compared to planar regions. The opposite is true for concave regions. For the case of textured TFTs with texturing along *θ* = 0, the channel comprises of flutes of concave, convex and planar regions running from source to drain. Since the convex regions accumulate free carriers at a lower gate voltage, they permit a current between source and drain much earlier as compared to the planar regions. Thus, the onset of conduction for textured TFTs with *θ* = 0 occurs at a lower gate voltage compared to conventional planar gate TFTs. In the case of textured TFTs with texturing along *θ* = *π*/2, the channel comprises of flutes of concave, convex and planar regions running along the channel width. Traversing from source to drain, the convex regions having carriers with larger *N*_*f*_ and *μ* would be interspersed with concave regions having low *N*_*f*_ and *μ* akin to a series of high and low resistances. There will therefore be no significant current established in the TFT till the concave regions also achieve a large enough *N*_*f*_. Thus, textured TFTs with lower *θ* begin conducting at lower gate voltage. Experiments show a small but definitive trend in this regard as observed in Fig. 4b for *L* = 40 *μ*m. *V*_*gs*_ at the onset of conduction for other channel lengths are given as Fig. S8 in the Supporting Information.

#### Impact on Contact Resistance

The contact resistance of the textured and planar gate TFTs was extracted from the intercept of plot of *V*_*ds*_/*I*_*ds*_ versus channel length. This measured value of contact resistance is plotted in Fig. 4c as a function of *θ* for textured TFTs (solid black) and for the planar gate TFTs (dashed blue) with the analytical model also shown (red). The contact resistance for textured TFTs with *θ* = 0 is seen to be lower than for the planar gate TFTs. The opposite is true for textured TFTs with *θ* = *π*/2. In general, there appears to be a gradual trend of increasing contact resistance as *θ* is increased. The exact mechanics for how the contact resistance is modulated by texturing is not very clear at this point. However, since the texturing extends below the source drain electrodes, the reasons could be similar to the manner by which the onset of conduction is modulated. This variation in contact resistance with texturing also explains the dependence of the relative strengths of the TFTs with channel length as observed in Fig. 2.

#### Current Voltage (I–V) Characteristics

We define the drain to source current for textured TFTs having texturing along *θ* to be *I*_*dθ*_ where the subscript *θ* takes the appropriate value for any specific case. It can be shown to be (see Supporting Information),

5 $$\begin{array}{c}{I}_{d\theta }=\frac{{\mu }_{0}\gamma }{(\alpha +\mathrm{1)(}{\varepsilon }_{i}/{t}_{i}{)}^{\nu }}({\int }_{0}^{{W}_{\theta }}{({\int }_{0}^{{L}_{\theta }}{c}_{i}^{-\alpha -\nu }{\rm{d}}x)}^{-1}{\rm{d}}y)({({V}_{gs}-{V}_{on}-{I}_{d\theta }{R}_{d\theta })}^{\alpha +1}\\ \,\,\,\,\,\,\,\,\,\,-{({V}_{gs}-{V}_{on}-{V}_{ds}+{I}_{d\theta }{R}_{d\theta })}^{\alpha +1})\end{array}$$

A more usable form of this model can be developed by making specific assumptions. First for strong above threshold operation, we set *α* = 1. Second, the variation in mobility with *c*_*i*_ is considered to be minimal and *ν* = 0. Third, the term *V*_*on*_ which represents the threshold voltage for large gate voltages is a constant. Fourth, for gentle texturing, *W*_*θ*_ ~ *W* and *L*_*θ*_ ~ *L*. Using these approximations,

6 $${I}_{d\theta }={\mu }_{0}\gamma ({\int }_{0}^{W}{({\int }_{0}^{L}{c}_{i}^{-1}{\rm{d}}x)}^{-1}{\rm{d}}y)({V}_{gs}-{V}_{on}-{V}_{ds}\mathrm{/2)(}{V}_{ds}-2{I}_{d\theta }{R}_{d\theta })$$

Figure 4d compares the experimentally obtained I–V characteristics for the textured TFT with texturing along different *θ* with the analytical model of Eq. (6). The model is seen to fit remarkably well with the data.

In addition to the purely analytical model of Eq. (6), it is also possible to predict *I*_*dθ*_ and the TFT parameters for any *θ* via an intuitive semi-empirical model. Noting the trends in TFT parameters with *θ* as seen in Fig. 3, it is possible to imagine these parameters defining an ellipse. Using this geometric intuition, it is possible to construct a semi-empirical model that defines the value of the drain-source current and all other TFT parameters for any *θ* using an elliptical function with the knowledge of their values at *θ* = 0 and *θ* = *π*/2. In other words, the value of these parameters at these points would represent the semi-major and semi-minor axis of the ellipse that could then be plotted on the polar plot of Fig. 3. By this definition,

7 $${I}_{d\theta }=\frac{{I}_{d0}{I}_{d\frac{\pi }{2}}}{{{(({I}_{d0}\sin (\theta ))}^{2}+{({I}_{d\frac{\pi }{2}}\cos (\theta ))}^{2})}^{1/2}}$$

The values of *I*_*d*0_ that would represent the semi-major axis of the ellipse and *I*_*dπ/2*_ that would represent the semi-minor axis of the ellipse can be defined from Eq. (6) to be

8 $$\begin{array}{c}{I}_{d0}={\mu }_{0}\gamma \frac{{\int }_{0}^{W}{c}_{i}{\rm{d}}y}{L}({V}_{gs}-{V}_{on}-{V}_{ds}\mathrm{/2)(}{V}_{ds}-2{I}_{d0}{R}_{d0})\\ {I}_{d\frac{\pi }{2}}={\mu }_{0}\gamma \frac{W}{{\int }_{0}^{L}{c}_{i}^{-1}{\rm{d}}x}({V}_{gs}-{V}_{on}-{V}_{ds}\mathrm{/2)}({V}_{ds}-2{I}_{d\frac{\pi }{2}}{R}_{d\frac{\pi }{2}})\end{array}$$

#### Impact on the Effective Capacitance per Unit Area, *C*_*i−eff*_

The definition of the local capacitance per unit area, *c*_*i*_(*x*, *y*) is valid for the elemental section d*x* by d*y*. However, the TFT properties are defined by the cumulative effect of *c*_*i*_ for all elemental section considered in the channel. We define the total effective capacitance per unit area as *C*_*i*−*eff*_. An accurate estimate of *C*_*i*−*eff*_ is obtained from Eq. (5) and Eq. (6) where

9 $$\begin{array}{rcl}{C}_{i-eff} & = & \frac{L}{W}({\int }_{0}^{{W}_{\theta }}{({\int }_{0}^{{L}_{\theta }}{c}_{i}^{-\alpha -\nu }{\rm{d}}x)}^{-1}{\rm{d}}y)\\  &  & \sim \frac{L}{W}({\int }_{0}^{W}{({\int }_{0}^{L}{c}_{i}^{-1}{\rm{d}}x)}^{-1}{\rm{d}}y)\end{array}$$

In general, defining the convex to have a smaller radius of curvature as compared to the concave regions would help increase *C*_*i*−*eff*_. If however, the convex and concave regions have the same radius of curvature, or if the radius of curvature of the concave region is smaller than the convex region, it is very possible that *C*_*i*−*eff*_ < *ε*_*i*_/*t*_*i*_, i.e. less than the effective capacitance per unit area of a planar gate TFT. For example, if the texturing consists of periodic convex and concave regions with the semiconductor-insulator interface having radius of curvature *r*_*c*_ in both regions, the effective capacitance in one spatial period would scale as ~(*ε*_*i*_/*r*_*c*_)((ln(1 + (*t*_*i*_/*r*_*c*_)))^−1^ + (ln(1/(1 − (*t*_*i*_/*r*_*c*_))))^−1^) and can be shown to be slightly less than *ε*_*i*_/*t*_*i*_. This also happens to be the case in the experiments of Fig. 2. Figure 4e shows the capacitance-voltage (C-V) characteristics obtained from textured and planar TFTs. The effective capacitance of the textured TFTs is seen to be slightly lower than the case of the planar gate TFTs. This is also corroborated by the results seen in Fig. 2. In large channel length TFTs, the relative importance of the channel resistance is more than the contact resistance. On the other hand, in short channel length TFTs, the channel resistance is much smaller and the contact resistance becomes more important. Hence the ratio of the above threshold current of the textured TFTs to the above threshold current of the planar gate TFT decreases with increasing channel length.

#### Impact on Sub-threshold Swing

The sub-threshold swing for the TFT is proportional to ln(10)*V*_*tc*_(1 + *C*_*t*_/*C*_*i*−*eff*_) with *C*_*t*_ being an effective capacitance of trap state. The sub-threshold swing is therefore modulated by *C*_*i*−*eff*_. From the C-V characteristics of Fig. 4e it is seen that *C*_*i*−*eff*_ is slightly lower than *ε*_*i*_/*t*_*i*_. Therefore, it is expected that the planar gate TFTs will have a slightly lower sub-threshold swing (and higher sub-threshold slope) as compared to the textured TFTs as corroborated by Fig. 3.

#### Impact on the Threshold Voltage

The effective threshold voltage of the TFT is best defined as *V*_*fb*_ + *qN*_*t*_/*C*_*i*−*eff*_ with *N*_*t*_ being the trap carrier concentration per unit area. From the C-V characteristics of Fig. 4e it is seen that *C*_*i*−*eff*_ is slightly lower than *ε*_*i*_/*t*_*i*_. Therefore, it is expected that the planar gate TFTs will have a slightly lower threshold voltage as compared to the textured TFTs (Corroborated by Fig. S6, Supplementary Information).

### Application: Voltage Amplifiers with Textured TFTs

We now address the example stated in the introduction to this work and consider the design of a common source amplifier of a certain gain *G* using TFTs based on a non-complementary process. If only planar gate TFTs are used, the desired gain can be achieved by ensuring the ratio of the aspect ratios of the driver TFT to the load TFT is *G* ^2^. We explore the possibility of boosting the gain not by the pure scaling of the aspect ratios but instead by also using the fact that the effective ratio of transconductance of the textured TFTs with the planar gate TFTs vary with *θ*.

**Figure 5:**
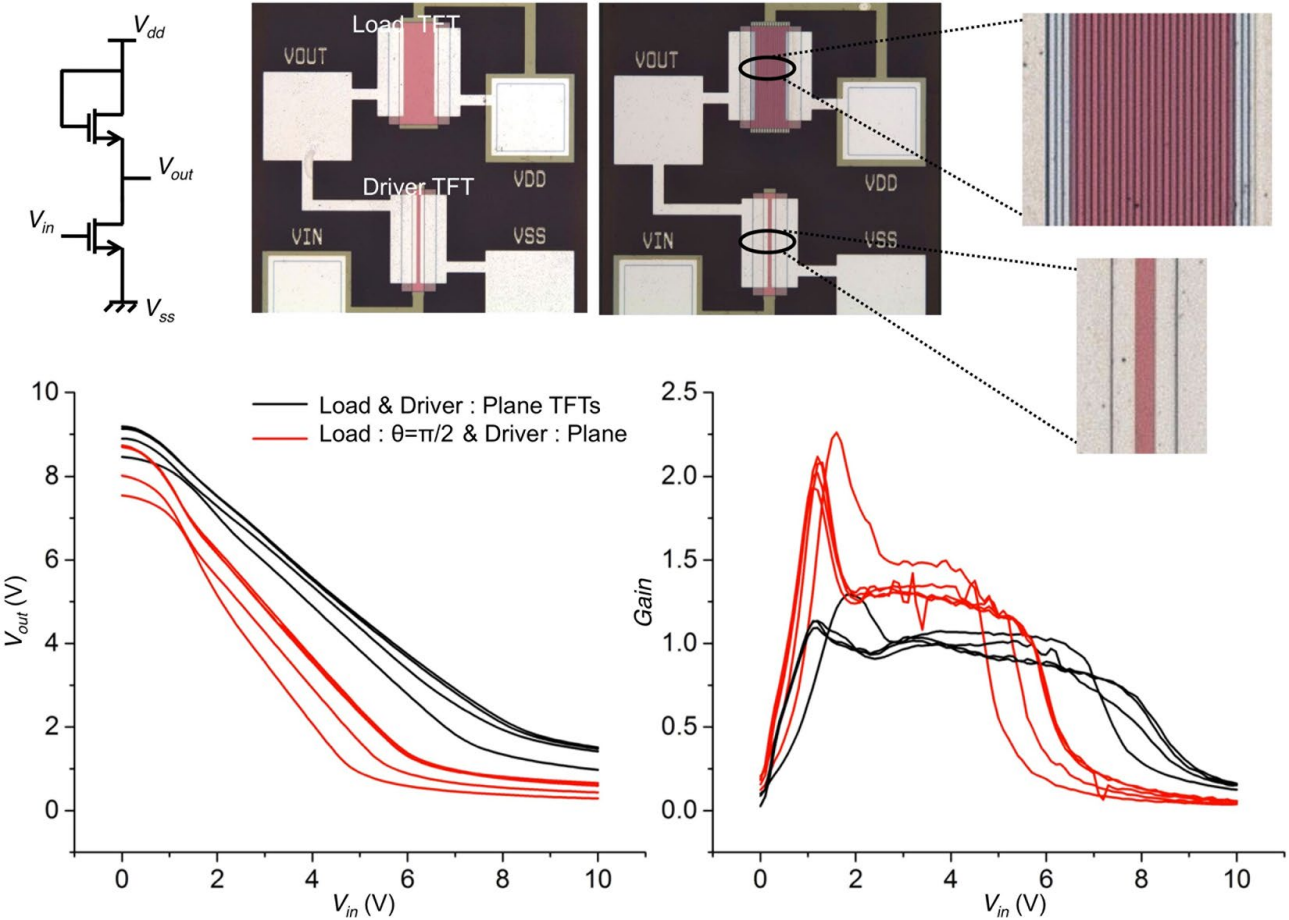
Textured TFTs can be used to boost the dc gain of voltage amplifiers without changing the layout area. Common source amplifiers with a driver and load element having a projected aspect ratio of 400/15 and 400/135, respectively were tested. In the first case, both the load and driver TFT had conventional planar gate architectures. This configuration yielded a dc gain of 1. In the second case, the drive TFT had a planar gate architecture while the load TFT was textured with periodic striated texturing along the *θ* = *π*/2 direction. This configuration showed a boosted dc gain of 2.

Figure 5 shows the schematic, micro-graphs and experimental results of two categories of amplifiers investigated. First, common source amplifiers based on the planar gate TFT with aspect ratio of 400/15 for the driver and 400/135 for the load were tested. The theoretically expected dc gain from this amplifier configuration is 3 while although a gain of about 1 was experimentally observed. The mismatch is attributed to the influence of contact resistance in the driver TFT. Next, amplifiers based on the planar gate TFT for the driver with aspect ratio 400/15 along with a textured gate TFT with aspect ratio 400/135 and with texturing along *θ* = *π*/2 for the load were tested. A dc gain of 2 was obtained. This boost in gain is achieved without altering the effective layout area of the circuit.

## Conclusion

This work studied the possibility of controlling the TFT transconductance via the texturing of the gate metal with specific geometric patterns. The texturing was accomplished via a dual gate metal deposition, the upper deposited metal being patterned. Specific to this study were textures based on periodic striations oriented along an angle *θ* with the direction of the channel length.

Experiments were performed with a-Si:H TFTs to identify the impact of texturing on the TFT I–V characteristics and TFT parameters. It was experimentally observed that textured TFTs with *θ* = 0 have a larger above threshold current, higher transconductance, quicker onset of conduction and smaller contact resistance as compared to textured TFTs with *θ* = *π*/2. A continuous trend was observed in all these parameters as *θ* was increased from 0 to *π*/2. Textured TFTs with *θ* = 0 also showed larger above threshold current (upto 40%) as compared to planar gate TFTs when the channel lengths were small. This is believed to be due to combined impact of the contact resistance and channel resistance. Textured TFTs with *θ* = *π*/2 consistently showed a lower above threshold current (upto −40%) compared to planar gate TFTs. The subthreshold slope and effective gate capacitance of planar gate TFTs were observed to be consistently and slightly higher than textured TFTs.

These observations could be explained via analytical models. The key results from the analysis can be summarized as follows. First, texturing results in a modulation of the local radius of curvature, *r*_*c*_, defined for any location (*x*, *y*) with *x* in the direction of the channel length and *y* in the direction of the channel width. The modulation in *r*_*c*_ causes a modulation in *c*_*i*_, the local gate capacitance per unit area defined for the elemental section d*x*d*y*. This in turn resulted in the modulation of the local surface potential *φ*_*s*_ and the local free carrier concentration per unit area, *N*_*f*_ with both *φ*_*s*_ and *N*_*f*_ being larger in convex regions as compared to concave regions. This explains the quicker turn on of textured TFTs with *θ* = 0 since the convex regions accumulate carrier earlier than the planar regions thereby providing a current path from source to drain at a lower gate voltage. On the other hand for the case of *θ* = *π*/2, the concave regions must also accumulate carriers before a current path exists between the source and drain. Second, lower contact resistance coupled with the above described behavior results in a higher above threshold current in short channel textured TFTs with *θ* = 0 as compared to planar gate TFTs. Third, the cumulative effect of the modulations in *c*_*i*_ results in the effective capacitance per unit area for the TFT, *C*_*i*−*eff*_, being slightly lower than *ε*_*i*_/*t*_*i*_. This explains the lower sub-threshold slope of the textured TFTs as compared to the planar gate TFTs.

Texturing therefore appears to provide an additional and strong control on the TFT parameters alongside aspect ratio and gate voltage based control. Transconductance modulation via texturing instead permits the use of minimum aspect ratio devices while retaining the ability to modulate transconductance by modulating *θ*. Compared to control via aspect ratio alone, this approach helps improve spatial resolution and performance as demonstrated by the design of the voltage amplifier. From the point of view of fabrication, texturing is readily adapted to fabrication methods such as ink-jet printing.

## Methods

### a-Si:H Thin Film Transistor Fabrication

Corning glass slide (Corning-2947, 75 mm × 50 mm), pre-cleaned using standard Piranha solution, was used as the substrate for device fabrication. The gate of the transistor was a 200 nm chromium metal layer deposited using e-beam evaporation and patterned using optical lithography. In order to create the striations, an additional 200 nm aluminum metal layer was deposited and patterned using photo-lithography. This was followed by the deposition of 200 nm silicon nitride as gate dielectric, 120 nm amorphous hydrogenated silicon (a-Si:H) as the active layer and 30 nm n-doped amorphous silicon (a-Si) as contact layer, all of which were deposited using plasma enhanced chemical vapor deposition. Subsequently, the active layer (a-Si:H) was patterned to isolate the devices and the insulator layer was patterned to open vias. Before source/drain electrode metal deposition, the substrate was dipped in dilute hydrofluoric acid for 10 s in order to remove any native oxide on the a-Si:H surface. The source-drain metal with 200 nm of Cr was deposited using e-beam evaporation and was patterned by lithography. Finally the n-doped a-Si layer was patterned and etched away from regions over the channel. All devices were designed to have a channel width of 400 *μm*. The striations had a height of 200 *nm*, a width of 4 *μm* and a pitch of 12 *μm*. The devices were annealed at 150 degrees for 1 hour in ambient conditions before testing.

### Measurement of I-V Characteristics

The TFT testing was carried out using Keithley 4200 Semiconductor Characterization System inside a probe station. The source, drain and gate pads of the devices were probed using tungsten probe tips, and were connected to three SMUs (Source Measure Units) of the Keithley through triax cables. For transfer characteristics, the drain voltage was stepped from 0 to 2 V in steps of 0.5 V. For each step, the gate voltage was swept from 0 to 10 V in steps of 0.1 V. The source SMU was set at a constant bias of 0 V. The compliance current for all SMUs was 1 mA. For output characteristics, the voltages were interchanged; the gate voltage was stepped from 0 to 10 V in steps of 2.5 V and for each step, the drain voltage was swept from 0 to 10 V in steps of 0.1 V. For amplifier testing, the four SMUs of the Keithley were connected to the *V*_*dd*_, *V*_*ss*_, *V*_*in*_ and *V*_*out*_ pads of the circuit. While *V*_*dd*_ and *V*_*ss*_, terminals were biased at constant voltages of 10 V and 0 V, the *V*_*in*_ SMU was swept from 0 to 10 V, in steps of 0.1 V and the *V*_*out*_ SMU was used as a voltmeter to measure the output voltage of the amplifier.

### Measurement of Capacitance-Voltage (C-V) Characteristics

The C-V measurement was carried out using Keithley 4200 Semiconductor Characterization System. Cable compensations were carried out to minimize any stray capacitances. The source/drain overlap capacitance to the gate was used for C-V measurement. A dc bias varying from−15V to +15 V along with a 10 kHz, 10 mV sinusoidal signal was applied between the two SMUs to obtain the C-V characteristics.

### Simulation Details

To analyze the variations in potential in the convex, concave and planar regions, ATLAS TCAD simulations on cylindrical metal-insulator-semiconductor capacitors were designed for simulation. These were akin to metal-insulator-semiconductor capacitors experiencing tensile or compressive bending. Both the concave and convex geometries were made with 2-D polar coordinates and the planar structure with 2-D rectangular coordinates. The insulator and the semiconductor were set to be SiN and intrinsic a-Si respectively with the thickness of 200 nm and 150 nm. The gate metal was chosen to be aluminum with a work-function of 4.1 eV. The radius of curvature (*r*_*c*_) for the bent geometries was chosen as 1 *μ*m. The arc length (in case of the planar structure, the length) of the semiconductor insulator interface was 3 *μm*. The effective density of states at the conduction band and the valence band edge of a-Si were chosen to be 2.5e20/cc and 2.5e20/cc, respectively. The band-gap and the electron-affinity of the a-Si were used as 1.8 *eV* and 3.8 *eV* respectively. The dielectric constant of SiN and a-Si were 7.5 and 11.8 respectively. The localized tail states and the deep states were modeled using the exponential distribution and the gaussian distribution, respectively. The characteristic energy and the density of states at the conduction band edge of acceptor-like tail states were 28 meV and 1e22/cc.eV, respectively. The total density of states, the characteristic decay energy and the peak energy location (with respect to the conduction band edge) of acceptor-like deep states were 1.5e15/cc.eV, 0.15 eV and 0.62 eV, respectively.

## Electronic supplementary material

Supplementary Information
