# Supplementary material for: Modulating Thin Film Transistor Characteristics by Texturing the Gate Metal
Source: Sci Rep. 2017 Dec 20;7:17932. doi: 10.1038/s41598-017-18111-5 (PMC5738405; doi:10.1038/s41598-017-18111-5)
Supplement: Supplementary file 1 — Supplementary Information [file 41598_2017_18111_MOESM1_ESM.pdf]

Modulating Thin Film Transistor Characteristics by Texturing the Gate Metal

Supplementary Information

Aswathi Nair [1], Prasenjit Bhattacharya[1] and Sanjiv Sambandan [1,2,*]

1. Indian Institute of Science, Department of Instrumentation and Applied Physics, Bangalore, 560012, India

2. University of Cambridge, Department of Engineering, Cambridge, CB3 0FF, United Kingdom

∗ sanjiv@iisc.ac.in

December 11, 2017

SEM images demsonstrating the conformality of metal-insulator-semiconductor stack

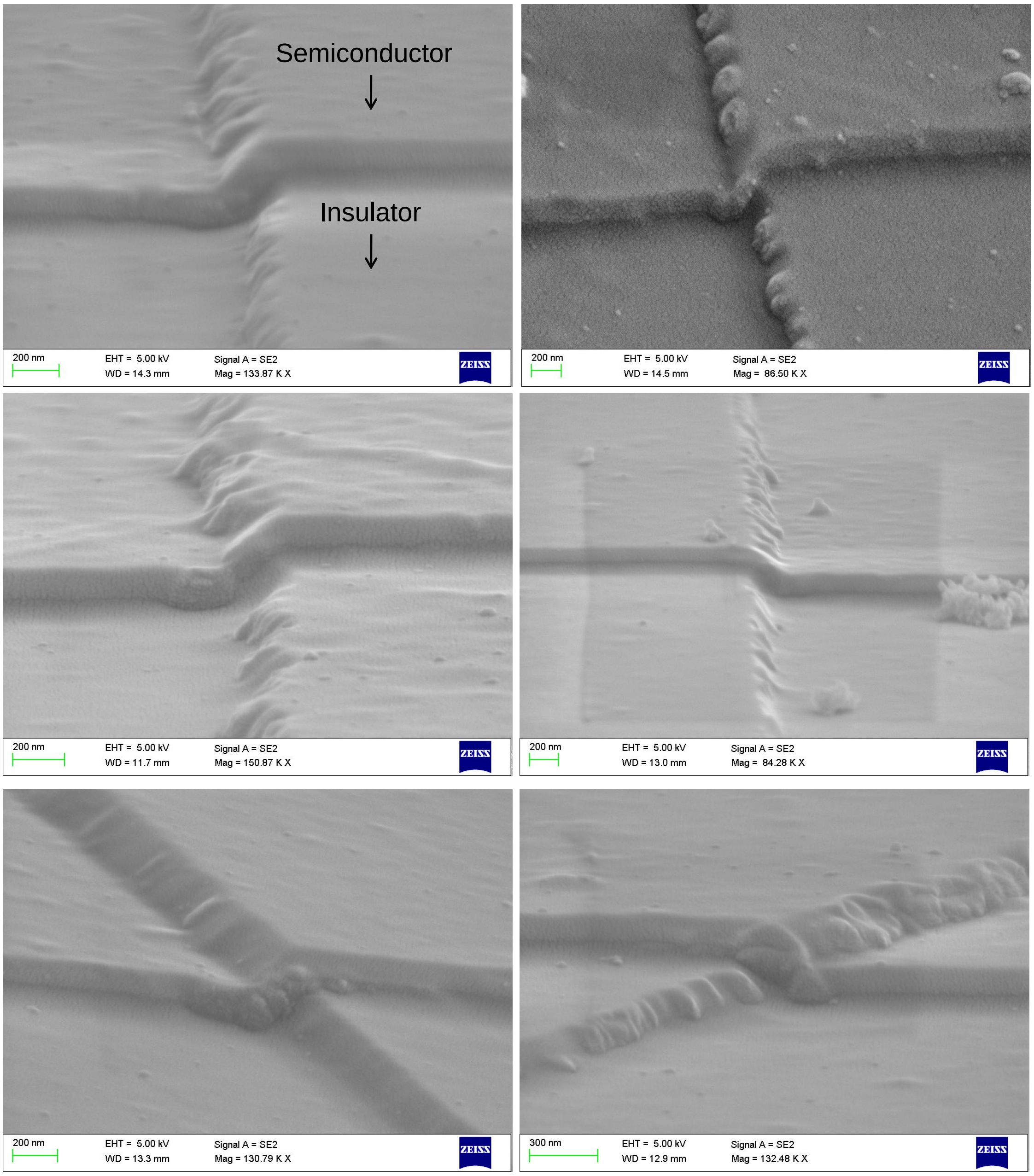

Figure S1: Scanning electron microscopy (SEM) images of the cross-section of the metal-insulator-semiconductor stack.

Detailed sets of device characteristics

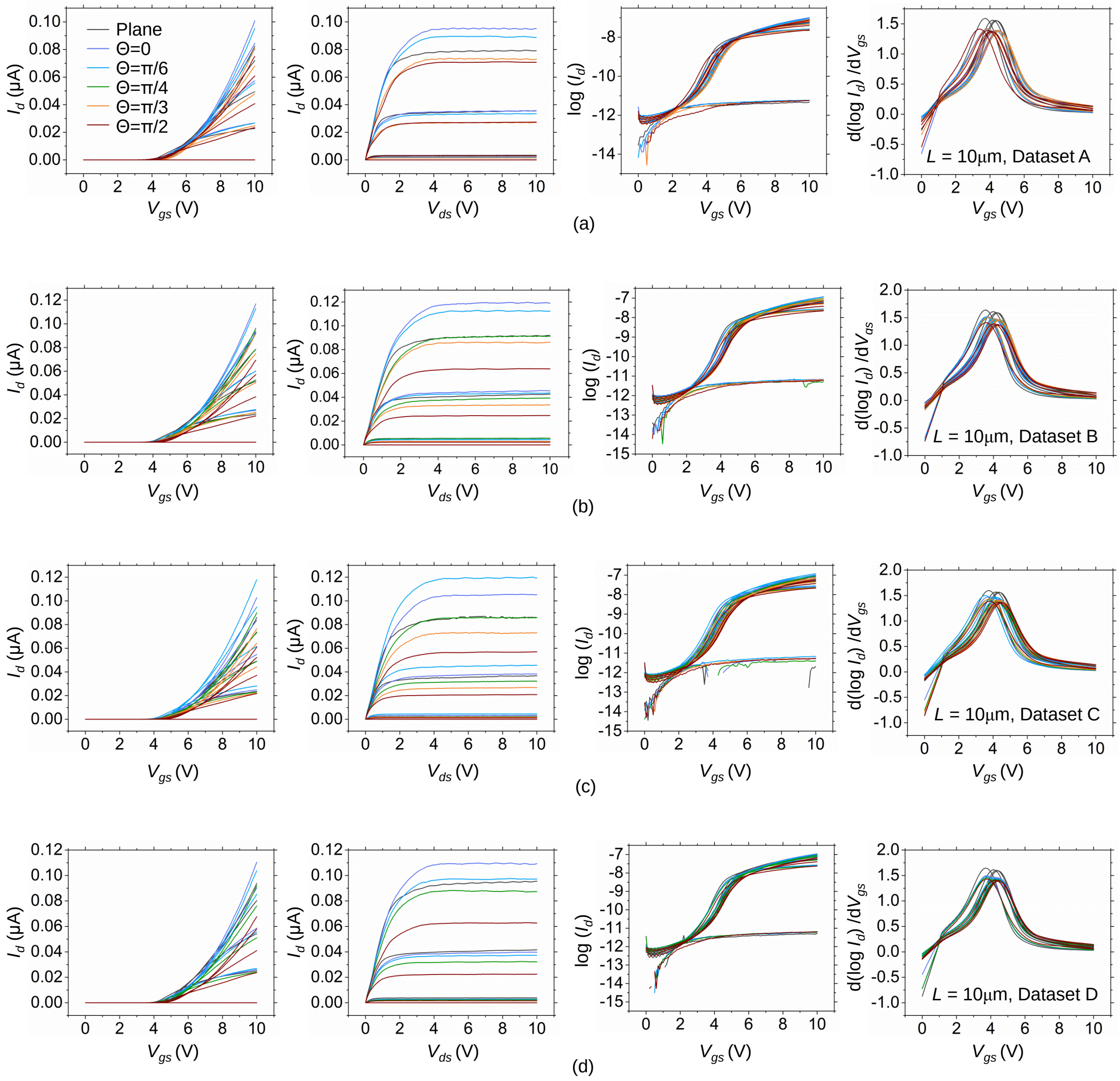

Figure S2: 4 datasets for plane and textured gate devices with channel length $L = 10\mu m$, each set consisting of transfer characteristics at V_{ds}=0 to 2V in steps of 0.5V, output characteristics at V_{gs}=0 to 10V in steps of 2.5V, transfer characteristics in log scale, derivative of transfer characteristics in log scale

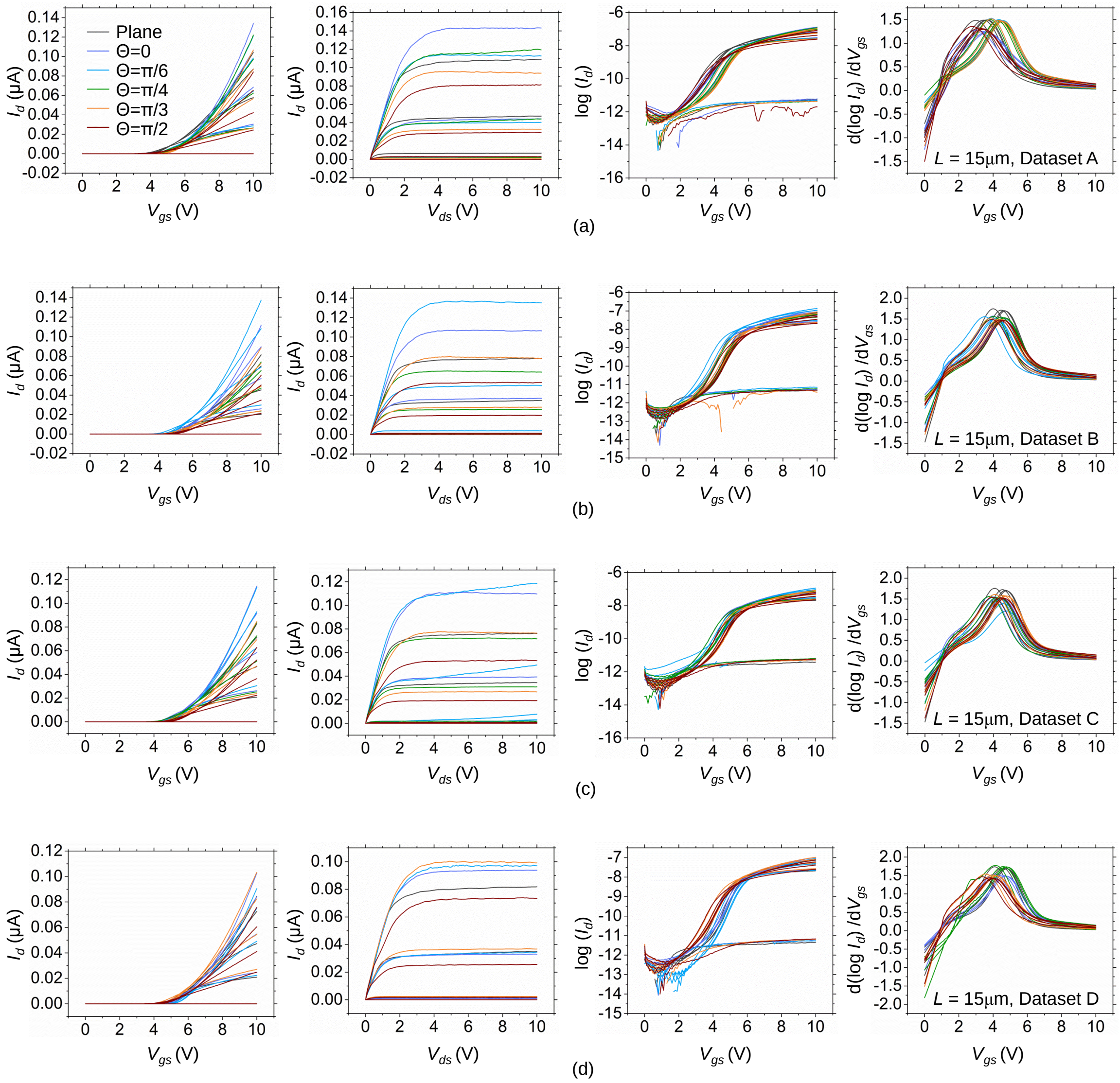

Figure S3: 4 datasets for plane and textured gate devices with channel length $L = 15\mu m$, each set consisting of transfer characteristics at V_{ds}=0 to 2V in steps of 0.5V, output characteristics at V_{gs}=0 to 10V in steps of 2.5V, transfer characteristics in log scale, derivative of transfer characteristics in log scale

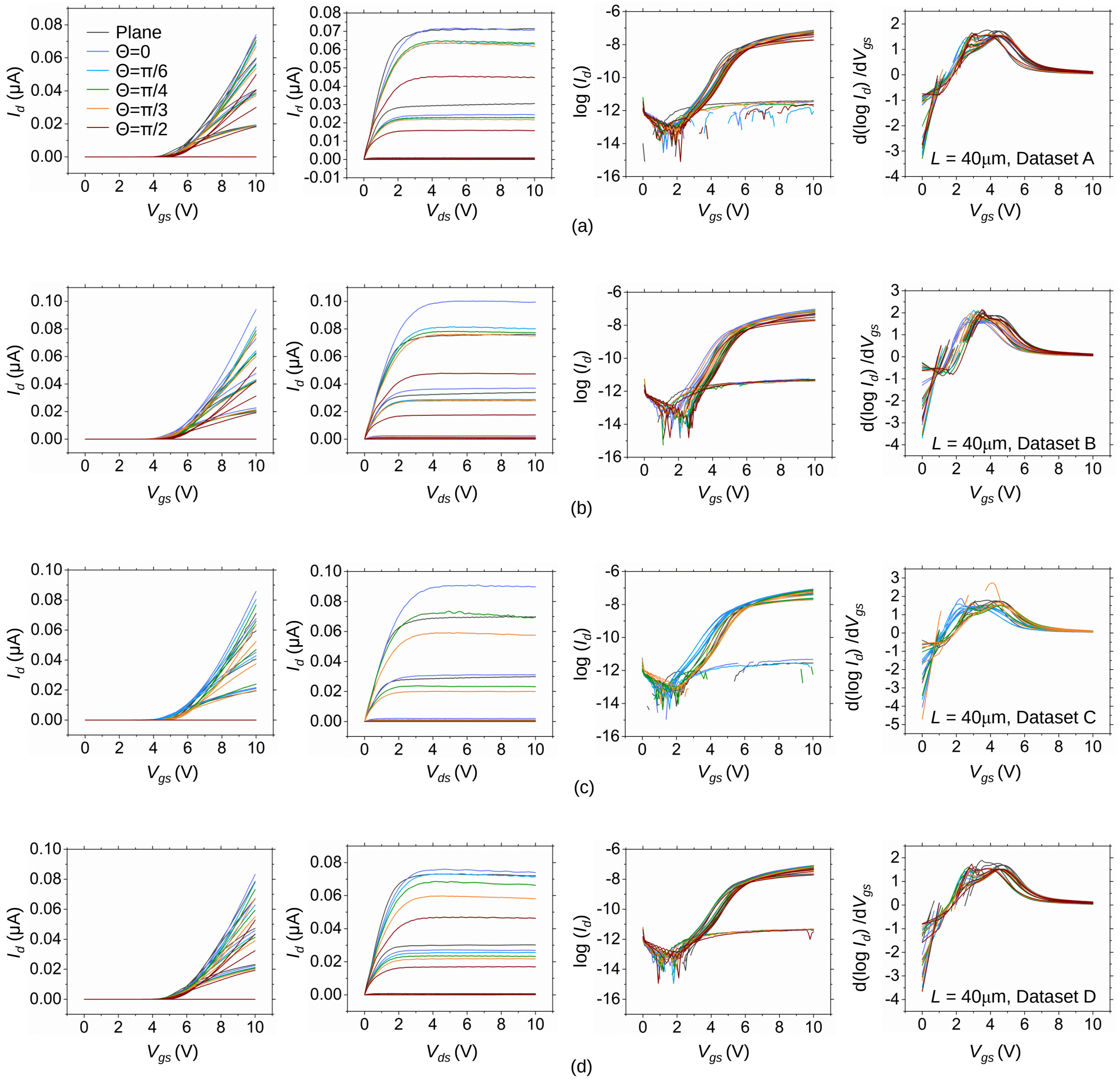

Figure S4: 4 datasets for plane and textured gate devices with channel length $L = 40\mu m$, each set consisting of transfer characteristics at V_{ds}=0 to 2V in steps of 0.5V, output characteristics at V_{gs}=0 to 10V in steps of 2.5V, transfer characteristics in log scale, derivative of transfer characteristics in log scale

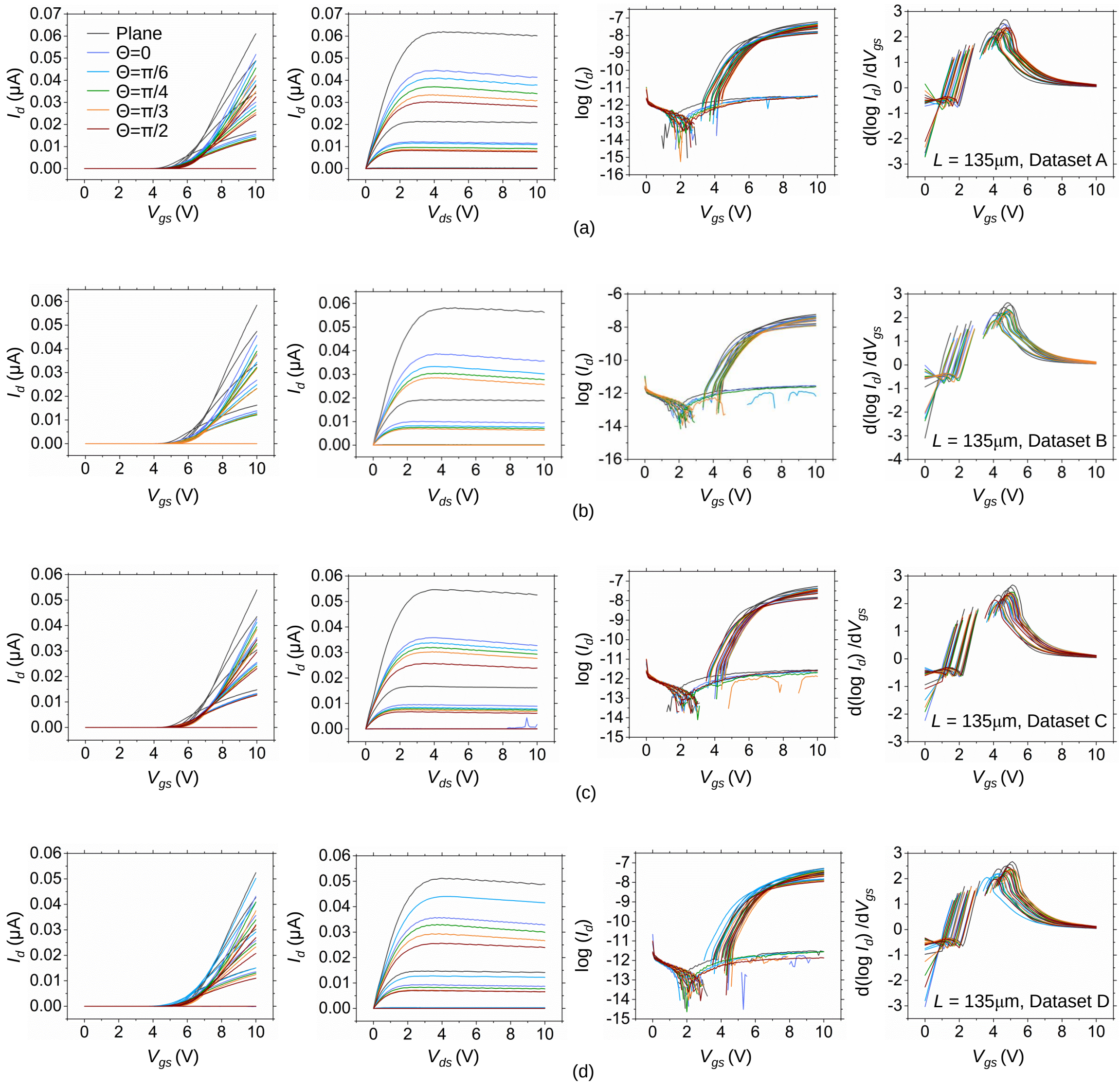

Figure S5: 4 datasets for plane and textured gate devices with channel length $L = 135\mu m$, each set consisting of transfer characteristics at V_{ds}=0 to 2V in steps of 0.5V, output characteristics at V_{gs}=0 to 10V in steps of 2.5V, transfer characteristics in log scale, derivative of transfer characteristics in log scale

Variation of TFT parameters with the angle of texturing

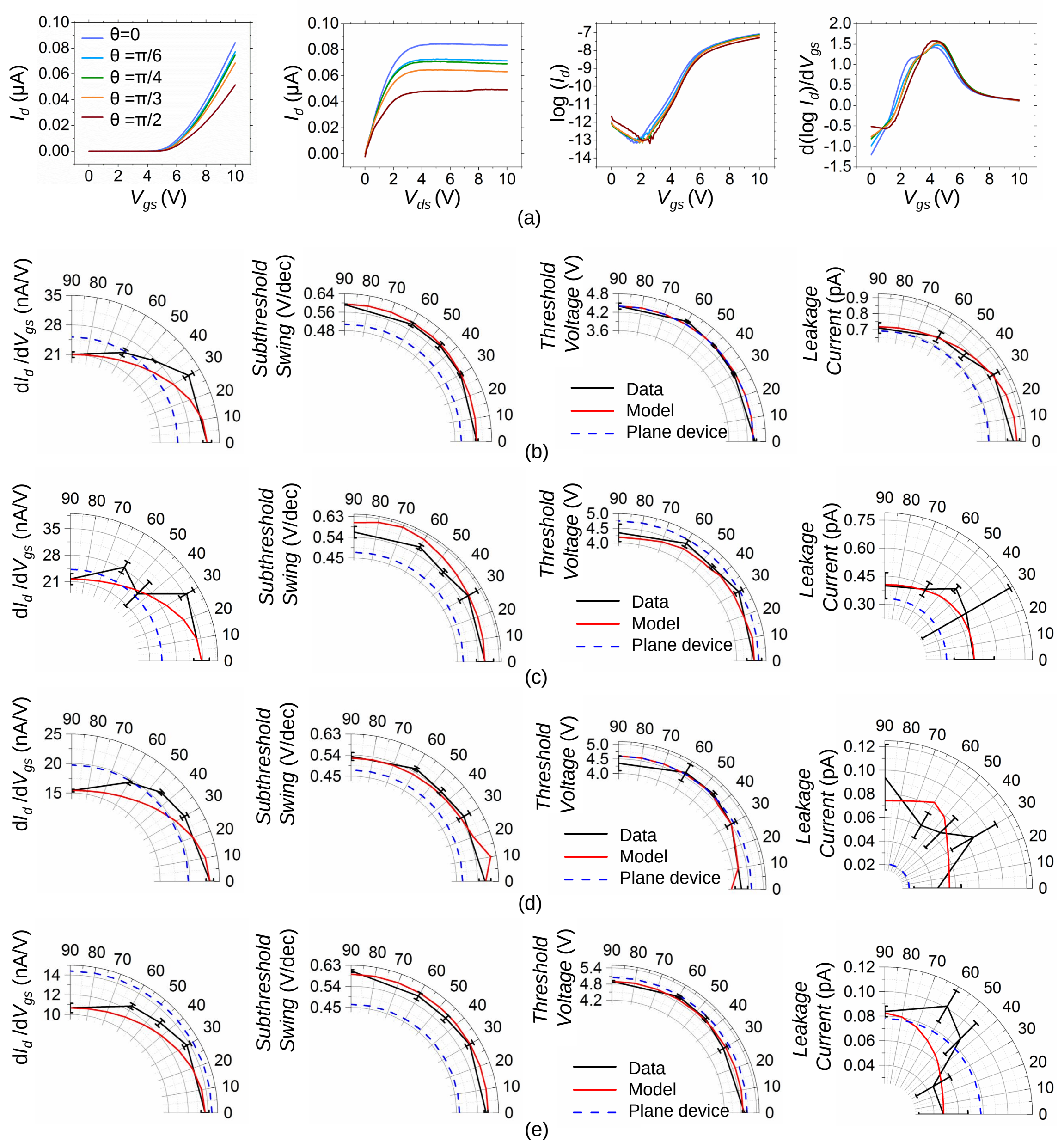

Figure S6: (a)I-V characteristics of textured TFTs with striated texturing along different θ. Transfer characteristics on a linear scale measured at $V_{ds} = 2$ V, output characteristics measured at $V_{gs} = 10$ V, transfer characteristics on a log scale and the plot of d$\log I_d$/dV_{gs} versus V_{gs} for channel length of 40 μm. Impact of θ on TFT parameters for channel lengths (b) $L = 10$ μ m, (c) $L = 15$ μm, (d) $L = 40$ μm and (e) $L = 135$ μm. Parameters extracted from experiment for textured TFTs (black solid line with markers), elliptical model (red solid line), corresponding parameter measured in planar gate TFT (blue dashed line)

Methods of extraction of various TFT parameters

All parameters shown in scatter plots are extracted from the transfer characteristics of the devices at V_{ds} = 2V.

1. dI_d/dV_{gs} : The slope of the transfer characteristics in the linear region (V_{gs} = 9.5V, V_{ds} = 2V).
2. Subthreshold swing : The inverse of peak value of the derivative of transfer characteristics in log scale.
3. Threshold voltage : The voltage at which dI_d/dV_{gs} curve reaches the maximum value.
4. Leakage current : The minimum value of I_d in log I_d vs V_{gs} plot.
5. V_{gs} at onset of conduction : The gate voltage at which I_d enters subthreshold region.

Co-ordinate system for planar and textured cases

The polar and Cartesian co-ordinates are interrelated.

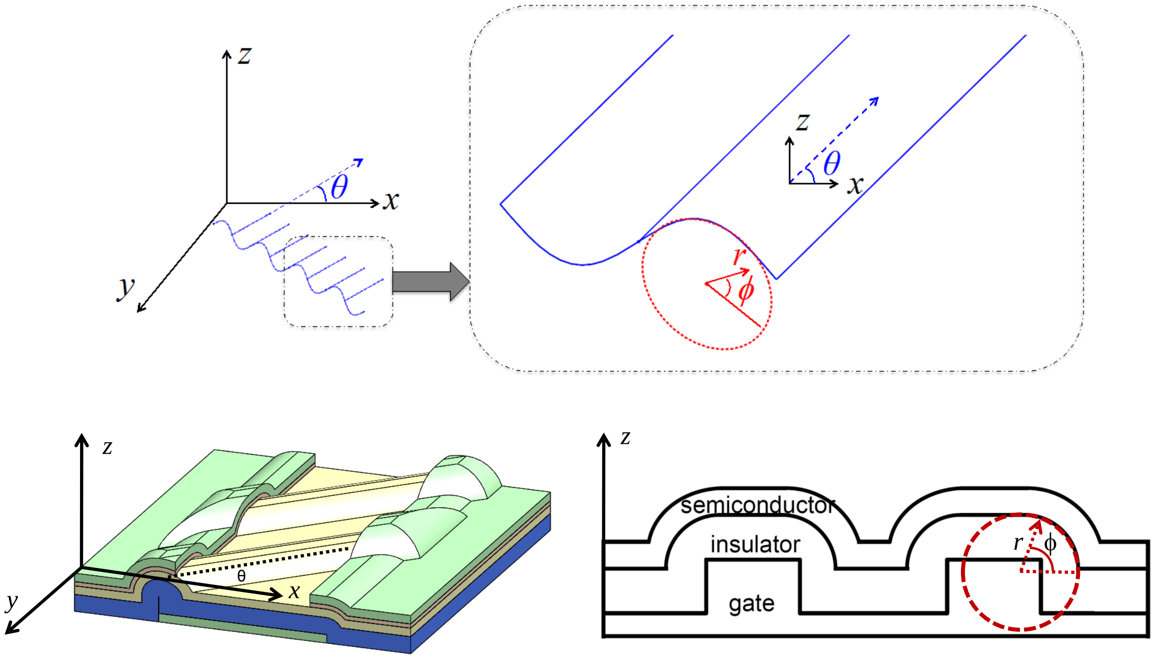

Figure S7: Illustration of the Co-ordinate system

For planar devices: There is only an $x - y$ coordinate system – along channel length and channel width, respectively. The z-axis normal to the semiconductor-insulator interface is not used.

For the textured device: We need the z-coordinate, but we prefer it in polar form (r, ϕ) with radial coordinate r, as it greatly simplifies the electrostatic analysis. The texturing is along a θ direction as shown in Fig. S7 (please note that this is not the angular coordinate of the polar system) Therefore this is the co-ordinate mapping for texturing:

$x = r\cos\phi\sin\theta$

$y = r\cos\phi\cos\theta$

$z = r\sin\phi$

Therefore, the $c_i(x, y)$ for an elemental section is also a $c_i(r, \phi)$.

Derivation of surface potential, free carrier concentration and I-V characteristics of textured gate TFTs

Solution to Poisson Boltzmann equation in polar coordinates

$$\frac{d^2\varphi}{dr^2} + \frac{1}{r}\frac{d\varphi}{dr} = \frac{qn_{t0}}{\epsilon_s}\, e^{\varphi/V_{tc}} \tag{1}$$

Let $\varphi/V_{tc} = p$

$$\frac{d^2p}{dr^2} + \frac{1}{r}\frac{dp}{dr} = \frac{qn_{t0}}{\epsilon_s V_{tc}}\, e^{p} \tag{2}$$

Let $\alpha = r^2 e^p$; $\beta = r\frac{dp}{dr}$,

$$\begin{aligned}\frac{d\beta}{d\alpha} = \frac{d\beta}{dr}\frac{dr}{d\alpha} &= \left(\frac{dp}{dr} + r\frac{d^2p}{dr^2}\right)\left(\frac{1}{2re^p + r^2e^p\frac{dp}{dr}}\right)\\ &= \left(\frac{1}{r}\frac{dp}{dr} + \frac{d^2p}{dr^2}\right)\left(\frac{r}{2re^p + r^2e^p\frac{dp}{dr}}\right)\end{aligned} \tag{3}$$

From Eqn. 2 and Eqn. 3,

$$\frac{d\beta}{d\alpha} = \frac{\left(\frac{qn_{t0}}{\epsilon_s V_{tc}}\right)e^p\, r}{2re^p + r^2e^p\frac{dp}{dr}} = \frac{\frac{qn_{t0}}{\epsilon_s V_{tc}}}{2+\beta} \tag{4}$$

Solving Eqn. 4,

$$2\beta + \frac{\beta^2}{2} = \frac{qn_{t0}}{\epsilon_s V_{tc}}\alpha - C_1 \qquad \text{where } C_1 \text{ - constant}$$

$$4\beta + \beta^2 - \frac{2qn_{t0}}{\epsilon_s V_{tc}}\alpha = -C_1$$

$$4r\frac{dp}{dr} + r^2\left(\frac{dp}{dr}\right)^2 - \frac{2qn_{t0}r^2e^p}{\epsilon_s V_{tc}} = -C_1 \tag{5}$$

Multiplying $2r^2$ to Eqn. 2,

$$2r^2\frac{d^2p}{dr^2} + 2r\frac{dp}{dr} - \frac{2qn_{t0}r^2e^p}{\epsilon_s V_{tc}} = 0$$

Subtract Eqn. 2 from Eqn. 5,

$$2r\frac{dp}{dr} - 2r^2\frac{d^2p}{dr^2} + r^2\left(\frac{dp}{dr}\right)^2 = -C_1 \tag{6}$$

Multiply $\frac{1}{r^2}\frac{dp}{dr}$ to Eqn. 6,

$$\frac{2}{r}\left(\frac{dp}{dr}\right)^2 - 2\frac{dp}{dr}\frac{d^2p}{dr^2} + \left(\frac{dp}{dr}\right)^3 = -\frac{C_1}{r^2}\frac{dp}{dr} \tag{7}$$

Eqn. 7 is modified to,

$$\frac{2}{r}\left(\frac{dp}{dr}\right)^2 - \frac{d\left(\left(\frac{dp}{dr}\right)^2\right)}{dr} + \left(\frac{dp}{dr}\right)^3 = -\frac{C_1}{r^2}\frac{dp}{dr}$$

Let $\left(\frac{dp}{dr}\right)^2 = z$, Therefore,

$$\frac{2}{r}z - \frac{dz}{dr} + z^{3/2} = -\frac{C_1}{r^2}z^{1/2}$$

Rewriting,

$$\frac{dz}{dr} = \frac{2}{r}z + z^{3/2} + \frac{C_1}{r^2}z^{1/2}$$

Using Wolfram symbolic differential equation solver, the exact solution for this is :

$$\begin{aligned} z(r) = \frac{1}{r^2}\Bigg[& C_1 \tan^2\left(\frac{1}{2}\sqrt{C_1 - 4}\ln(r) - \frac{1}{2}\sqrt{C_1 - 4}\; C_2\right) \\ & -4\tan^2\left(\frac{1}{2}\sqrt{C_1 - 4}\ln(r) - \frac{1}{2}\sqrt{C_1 - 4}\; C_2\right) \\ & -4\sqrt{C_1 - 4}\tan\left(\frac{1}{2}\sqrt{C_1 - 4}\ln(r) - \frac{1}{2}\sqrt{C_1 - 4}\; C_2\right) + 4\Bigg] \end{aligned}$$

$$z(r) = \frac{1}{r^2}\left[\sqrt{C_1 - 4}\tan\left(\frac{\sqrt{C_1 - 4}\ln(r) - \sqrt{C_1 - 4}\; C_2}{2}\right) - 2\right]^2$$

If $\sqrt{C_1/4 - 1} = \kappa$ and $C_2 = \ln l_b$,

$$z(r) = \frac{4}{r^2}\left[\kappa\tan\left(\kappa\ln\left(\frac{r}{l_b}\right)\right) - 1\right]^2 \tag{8}$$

Since $\left(\frac{dp}{dr}\right)^2 = z$,

$$\frac{dp}{dr} = \pm\frac{2}{r}\left[\kappa\tan\left(\kappa\ln\left(\frac{r}{l_b}\right)\right) - 1\right]$$

The solution with the - sign does not satisfy Eq. 2, therefore

$$p = 2\ln\left(\frac{l_c}{r}\sec\left(\kappa\ln\left(\frac{r}{l_b}\right)\right)\right) \tag{9}$$

Here the constant of integration has been absorbed in the logarithm term to generate a constant coefficient l_c. Substituting Eqn. 9 in Eqn. 2, we get $l_c = \kappa l_{tc}$ where $l_{tc} = \sqrt{2\epsilon_s V_{tc}/qn_{t0}}$. Therefore,

$$p = 2\ln\left(\frac{\kappa l_{tc}}{r}\sec\left(\kappa\ln\left(\frac{r}{l_b}\right)\right)\right) \tag{10}$$

$$\varphi(r) = 2V_{tc}\ln\left(\frac{\kappa l_{tc}}{r}\sec\left(\kappa\ln\left(\frac{r}{l_b}\right)\right)\right) \tag{11}$$

The boundary conditions to be used are,

$$\xi(r = r_c + t_s) = 0, \quad \varphi(r = r_c + t_s) = 0 \qquad for \quad concave \tag{12}$$

$$\xi(r = r_c - t_s) = 0, \quad \varphi(r = r_c - t_s) = 0 \qquad for \quad convex \tag{13}$$

$$c_{i\pm}(V_{gs} - V_{fb} - \varphi_s) = \pm\epsilon_s\xi(r = r_c), \quad + \, for \; concave \; and \; - \; for \; convex \tag{14}$$

According to the boundary conditions Eqn. 12 and Eqn. 13, we apply $\frac{dp}{dr} = 0$, $p = 0$ at $r = r_c \pm t_s$ in Eqn. 5 to obtain,

$$C_1 = \frac{2qn_{t0}(r_c \pm t_s)^2}{\epsilon_s V_{tc}} = 4(r_c \pm t_s)^2/l_{tc}^2$$

Therefore,

$$\kappa = \sqrt{C_1/4 - 1} = \sqrt{\left(\frac{r_c \pm t_s}{l_{tc}}\right)^2 - 1} \tag{15}$$

with the '+' and '-' signs used for concave and convex cases respectively.

Inorder to use the boundary condition given in Eqn. 14, we calculate the electric field (ξ) from Eqn. 11 to be,

$$\xi = -\frac{d\varphi}{dr} = -\frac{2V_{tc}}{r}\left(\kappa\tan\left(\kappa\ln\left(\frac{r}{l_b}\right)\right) - 1\right)$$

Substituting for 'tan $\left(\kappa\ln\left(\frac{r}{l_b}\right)\right)$' from Eqn. 11

$$\xi = -\frac{2V_{tc}}{r}\left(\pm\left(\frac{r}{l_{tc}}\left(e^{\varphi/V_{tc}} - \left(\frac{\kappa l_{tc}}{r}\right)^2\right)^{1/2}\right) - 1\right)$$

$$\xi = \frac{2V_{tc}}{l_{tc}}\left(\pm\left(e^{\varphi/V_{tc}} - \left(\frac{\kappa l_{tc}}{r}\right)^2\right)^{1/2} + \frac{l_{tc}}{r}\right) \tag{16}$$

In the boundary condition in Eqn. 14, the LHS represents the magnitude of charge at the insulator semiconductor interface, which is positive. We have used appropriate sign change on the RHS of the boundary condition to adapt to the geometric conventions that we have used. In our case r is positive in the radially outward direction, and ξ will be positive in concave case and negative with respect to r in convex case. Substituting the electric field expression in Eqn. 16 at $r = r_c$ in the boundary condition given in Eqn. 14 yields,

$$c_{i\pm}(V_{gs} - V_{fb} - \varphi_s) = \pm\frac{2V_{tc}\epsilon_s}{l_{tc}}\left(\pm\left(e^{\varphi_s/V_{tc}} - \left(\frac{\kappa l_{tc}}{r_c}\right)^2\right)^{1/2} - \frac{l_{tc}}{r_c}\right)$$

Also noting that $l_{tc} << r_c$,

$$c_{i\pm}(V_{gs} - V_{fb} - \varphi_s) = \frac{2V_{tc}\epsilon_s}{l_{tc}}\left(e^{\varphi_s/V_{tc}} - \left(\frac{\kappa l_{tc}}{r_c}\right)^2\right)^{1/2}$$

Using $l_{tc} << r_c$, κ can be approximated to be $(r_c \pm t_s)/l_{tc}$. Substituting for κ in the above expression yields,

$$V_{gs} - V_{fb} - \varphi_s = \frac{2V_{tc}\epsilon_s}{l_{tc}c_{i\pm}}\left(e^{\varphi_s/V_{tc}} - 1 \pm \frac{t_s}{r_c}\right)^{1/2}$$

Since $e^{\varphi_s/V_{tc}} >> 1 > t_s/r_c$, the above expression simplifies to,

$$V_{gs} - V_{fb} - \varphi_s = \frac{2V_{tc}\epsilon_s}{l_{tc}c_{i\pm}}e^{\varphi_s/2V_{tc}} \tag{17}$$

The Eqn. 17 is in the form $e^x = ax + b$, whose solution is given by the zero order Lambert W function as,

$$x = -\mathbf{W}_0\left(\frac{-1}{a}e^{-b/a}\right) - \frac{b}{a}$$

In this case, $a = -(l_{tc}c_{i\pm})/\epsilon_s$ and $b/a = -(V_{gs} - V_{fb})/(2V_{tc})$. Therefore, the surface potential for concave and convex TFTs is given by,

$$\frac{\varphi_s}{2V_{tc}} = \frac{V_{gs} - V_{fb}}{2V_{tc}} - \mathbf{W}_0\left(\frac{\epsilon_s}{l_{tc}c_{i\pm}}e^{(V_{gs}-V_{fb})/2V_{tc}}\right)$$

$$\varphi_s = V_{gs} - V_{fb} - 2V_{tc}\mathbf{W}_0\left(\frac{\epsilon_s}{l_{tc}c_{i\pm}}e^{(V_{gs}-V_{fb})/2V_{tc}}\right) \tag{18}$$

where '+' and '-' corresponds to concave and convex cases respectively. As Lambert W function can be approximated as $\mathbf{W}_0(x) = \ln(x) - \ln(\ln(x))$ at high V_{gs},

$$\varphi_s = V_{gs} - V_{fb} - 2V_{tc}\left[\ln\left(\frac{\epsilon_s}{l_{tc}c_{i\pm}}\right) + \frac{V_{gs} - V_{fb}}{2V_{tc}} - \ln\left(\ln\left(\frac{\epsilon_s}{l_{tc}c_{i\pm}}\right) + \frac{V_{gs} - V_{fb}}{2V_{tc}}\right)\right]$$

Neglecting $\ln(\epsilon_s/(l_{tc}c_{i\pm}))$ in the second term,

$$\varphi_s = -2V_{tc}\left[\ln\left(\frac{\epsilon_s}{l_{tc}c_{i\pm}}\right) - \ln\left(\frac{V_{gs}-V_{fb}}{2V_{tc}}\right)\right]$$

Using $l_{tc} = \sqrt{(2\epsilon_s V_{tc})/(qn_{t0})}$, the surface potential is given by,

$$\varphi_s = 2V_{tc}\ln\left(\frac{c_{i\pm}(V_{gs}-V_{fb})}{qn_{t0}l_{tc}}\right) \tag{19}$$

Here + corresponds to concave case and - corresponds to convex case. The insulator capacitance in a textured gate TFT will vary spatially depending on the curvature of the insulator semiconductor interface and will take values corresponding to planar, concave or convex cases, as discussed in the manuscript. Also, by referring all voltages to the source electrode, the surface potential can be expressed as

$$\varphi_s = V_{gs} - V_{fb} - 2V_{tc}\mathbf{W}_0\left(\frac{\epsilon_s}{l_{tc}c_i(x,y)}e^{(V_{gs}-V_{fb}-V_{ch})/2V_{tc}}\right) \tag{20}$$

$$\varphi_s = 2V_{tc}\ln\left(\frac{c_i(x,y)(V_{gs}-V_{fb}-V_{ch})}{qn_{t0}l_{tc}}\right) \tag{21}$$

In order to calculate l_b, use $\varphi(r-r_c) = \varphi_s$ in Eqn. 11,

$$e^{\varphi_s/2V_{tc}} = \left(\frac{\kappa l_{tc}}{r_c}\sec\left(\kappa\ln\left(\frac{r_c}{l_b}\right)\right)\right)$$

$$\kappa\ln\left(\frac{r_c}{l_b}\right) = \sec^{-1}\left(\frac{r_c}{\kappa l_{tc}}e^{\varphi_s/2V_{tc}}\right)$$

Since $\sec^{-1}$ can return both positive and negative values, we mention this explicitly by using a $\pm$ sign. Therefore,

$$l_b = r_c\exp\left(\frac{-1}{\kappa}\left(\pm\left|\sec^{-1}\left(\frac{r_c}{\kappa l_{tc}}e^{\varphi_s/2V_{tc}}\right)\right|\right)\right) \tag{22}$$

Using Eqn. 22 in Eqn. 11, we get the potential variation in the semiconductor to be,

$$\varphi(r) = 2V_{tc}\ln\left(\frac{\kappa l_{tc}}{r}\sec\left(\pm\left|\sec^{-1}\left(\frac{r_c}{\kappa l_{tc}}e^{\varphi_s/2V_{tc}}\right)\right| - \kappa\ln\left(\frac{r}{r_c}\right)\right)\right) \tag{23}$$

where + is for the concave case and - is for the convex case.

Calculation of Free Carrier Concentration, N_f

Electrons available for conduction in the semciconductor = N_f $(/cm^2)$

$$N_f = \int_0^{t_s} n_f\, dx$$

where t_s = thickness of semiconductor.

$$-d\varphi/dx = \xi \Rightarrow dx = -d\varphi/\xi$$
$$N_f = -\int_{\varphi_s}^{0} \frac{n_f}{\xi} d\varphi$$

Here $n_f = n_{f0}\, e^{\varphi/V_{th}}$ and ξ is calculated from Poisson's equation.

1. Planar Case

$$\frac{d^2\varphi}{dz^2} = \frac{q(n_t + n_f)}{\epsilon_s}$$

Case 1a: (during turn on)

$$n_t + n_f \cong n_t$$
$$\frac{d^2\varphi}{dz^2} \cong \frac{qn_{t0}}{\epsilon_s}\ e^{\frac{\varphi}{V_{tc}}}$$
$$2\frac{d\varphi}{dz}\frac{d^2\varphi}{dz^2} \cong \frac{2qn_{t0}}{\epsilon_s}\ e^{\frac{\varphi}{V_{tc}}}\ \frac{d\varphi}{dz}$$
$$\frac{d}{dz}\left(\left(\frac{d\varphi}{dz}\right)^2\right) \cong \frac{2qn_{t0}}{\epsilon_s}\ e^{\frac{\varphi}{V_{tc}}}\ \frac{d\varphi}{dz}$$

Integrating both sides,

$$\left(\frac{d\varphi}{dz}\right)^2 = \xi^2 \cong \frac{2qn_{t0}V_{tc}}{\epsilon_s}\ e^{\frac{\varphi}{V_{tc}}} + const$$

Using the boundary condition,

$$\varphi = 0, \xi = 0 \Rightarrow const = -\frac{2qn_{t0}V_{tc}}{\epsilon_s}$$
$$\xi^2 = \frac{2qn_{t0}V_{tc}}{\epsilon_s}\left(e^{\frac{\varphi}{V_{tc}}} - 1\right)$$
$$\xi = \left[\frac{2qn_{t0}V_{tc}}{\epsilon_s}\left(e^{\frac{\varphi}{V_{tc}}} - 1\right)\right]^{\frac{1}{2}}$$
$$\xi \approx \left(\frac{2qn_{t0}V_{tc}}{\epsilon_s}\right)^{\frac{1}{2}}\ e^{\frac{\varphi}{2V_{tc}}}$$

Therefore,

$$N_f = -\int_{\varphi_s}^{0} \frac{n_{f0}\, e^{\frac{\varphi}{Vth}}}{\left(\frac{2qn_{t0}V_{tc}}{\epsilon_s}\right)^{\frac{1}{2}}\ e^{\frac{\varphi}{2V_{tc}}}}\ d\varphi$$

$$= -\int_{\varphi_s}^{0} \frac{n_{f0}\epsilon_s^{\frac{1}{2}}}{(2qn_{t0}V_{tc})^{\frac{1}{2}}} \; e^{\varphi\left(\frac{1}{V_{th}}-\frac{1}{2V_{tc}}\right)} \; d\varphi$$

$$= -\frac{n_{f0}\epsilon_s^{\frac{1}{2}}}{(2qn_{t0}V_{tc})^{\frac{1}{2}}} \left(\frac{1}{V_{th}} - \frac{1}{2V_{tc}}\right)^{-1} \; e^{\frac{\varphi}{2V_{tc}}\left(\frac{2V_{tc}}{V_{th}}-1\right)}\Big|_{\varphi_s}^{0}$$

$$\approx \frac{n_{f0}\epsilon_s^{\frac{1}{2}}}{(2qn_{t0}V_{tc})^{\frac{1}{2}}} \frac{2V_{tc}}{\left(\frac{2V_{tc}}{V_{th}} - 1\right)} \; e^{\frac{\varphi_s}{2V_{tc}}\left(\frac{2V_{tc}}{V_{th}}-1\right)}$$

Let $\alpha_{tc} = \frac{2V_{tc}}{V_{th}} - 1$. Noting that,

$$\varphi_s \approx 2V_{tc} \ln\left[\frac{c_i(V_{gs} - V_{fb} - V_{ch})}{qn_{t0}l_{tc}}\right]$$

$$N_f = \frac{n_{f0}}{\alpha} \frac{\sqrt{2V_{tc}\epsilon_s}}{\sqrt{qn_{t0}}} \frac{(c_i(V_{gs} - V_{fb} - V_{ch}))^{\alpha}}{(qn_{t0}l_{tc})^{\alpha}}$$

$$\Rightarrow N_f = \frac{n_{f0}l_{tc}}{\alpha(qn_{t0}l_{tc})^{\alpha}} \left(c_i(V_{gs} - V_{fb} - V_{ch})\right)^{\alpha}$$

Therefore,

$$qN_f = \gamma_{tc} \left(c_i(V_{gs} - V_{fb} - V_{ch})\right)^{\alpha}$$

$$\gamma_{tc} = \frac{1}{\alpha} \frac{n_{f0}}{(n_{t0})^{\alpha}} \frac{1}{(ql_{tc})^{\alpha-1}}$$

$$\gamma_{tc} = \frac{1}{\alpha} \frac{n_{f0}}{n_{t0}} \frac{1}{(\sqrt{2}q\epsilon_s V_{tc} n_{t0})^{(\alpha-1)/2}}$$

Case 1b:

$$n_t + n_f \cong n_f$$

$$\frac{d^2\varphi}{dz^2} \cong \frac{qn_{f0}}{\epsilon_s} \; e^{\frac{\varphi}{V_{th}}}$$

$$\xi \approx \left(\frac{2qn_{f0}V_{th}}{\epsilon_s}\right)^{\frac{1}{2}} \; e^{\frac{\varphi}{2V_{th}}}$$

Therefore,

$$N_f = -\int_{\varphi_s}^{0} \frac{n_{f0}\, e^{\frac{\varphi}{V_{th}}}}{\left(\frac{2qn_{f0}V_{th}}{\epsilon_s}\right)^{\frac{1}{2}} \; e^{\frac{\varphi}{2V_{th}}}} \; d\varphi$$

$$= -\frac{n_{f0}\epsilon_s^{\frac{1}{2}}}{(2qn_{f0}V_{th})^{\frac{1}{2}}} \, 2V_{th} \; e^{\frac{\varphi}{2V_{th}}}\Big|_{\varphi_s}^{0}$$

$$\approx n_{f0} \left(\frac{2V_{th}\epsilon_s}{qn_{f0}}\right)^{1/2} e^{\frac{\varphi_s}{2V_{th}}}$$

Noting that,

$$\varphi_s \approx 2V_{th} \ln\left[\frac{c_i(V_{gs} - V_{fb} - V_{ch})}{qn_{f0}l_{th}}\right]$$

$$\Rightarrow N_f = \frac{n_{f0}l_{th}}{(qn_{f0}l_{th})^{\alpha_{th}}} \left(c_i(V_{gs} - V_{fb} - V_{ch})\right)^{\alpha_{th}}, \qquad \alpha_{th} = 1$$

Therefore,

$$qN_f = \gamma_{th}\left(c_i(V_{gs} - V_{fb} - V_{ch})\right)^{\alpha_{th}}$$

$$\gamma_{th} = \frac{qn_{f0}l_{th}}{(qn_{f0}l_{th})^{\alpha_{th}}} \approx 1$$

2. Polar Coordinates

Depending on the capacitance of concave or convex cases, the free carrier concentration also varies, according to the equation,

$$qN_f = \gamma_{tc}\left(c_i(V_{gs} - V_{fb} - V_{ch})\right)^{\alpha}$$

$$\gamma_{tc\,polar} = \gamma_{tc\,planar}$$

I-V Characteristics

Current per unit width,

$$I_{d\theta/W} = qN_f(x,y)\mu(x,y)\frac{dV_{ch}}{dx}$$

Substituting for $qN_f = \gamma\left(c_i(V_{gs} - V_{on} - V_{ch})\right)^{\alpha}$ and $\mu = \mu_0\left(\frac{c_i t_i}{\epsilon_i}\right)^{\nu}$,

$$\begin{aligned}
I_{d\theta/W} &= \gamma\left(c_i(V_{gs} - V_{on} - V_{ch})\right)^{\alpha}\,\mu_0\left(\frac{c_i t_i}{\epsilon_i}\right)^{\nu}\frac{dV_{ch}}{dx} \\
&= \frac{\gamma\mu_0}{(\epsilon_i/t_i)^{\nu}}c_i^{\alpha+\nu}\left(V_{gs} - V_{on} - V_{ch}\right)^{\alpha}\frac{dV_{ch}}{dx} \\
\frac{I_{d\theta/W}}{c_i^{\alpha+\nu}}dx &= \frac{\gamma\mu_0}{(\epsilon_i/t_i)^{\nu}}(V_{gs} - V_{on} - V_{ch})^{\alpha}dV_{ch}
\end{aligned}$$

Integrating both sides,

$$\int_0^{L_\theta}\frac{I_{d\theta/W}}{c_i^{\alpha+\nu}}dx = \frac{\gamma\mu_0}{(\epsilon_i/t_i)^{\nu}}\int_0^{V_{ds}}(V_{gs} - V_{on} - V_{ch})^{\alpha}dV_{ch}$$

$$I_{d\theta/W} = \frac{\gamma\mu_0}{(\alpha+1)((\epsilon_i/t_i)^{\nu})}\left(\int_0^{L_\theta}c_i^{-\alpha-\nu}dx\right)^{-1}\left[(V_{gs} - V_{on})^{\alpha+1} - (V_{gs} - V_{on} - V_{ds})^{\alpha+1}\right]$$

Therefore, the total drain current is given by,

$$I_{d\theta} = \frac{\gamma\mu_0}{(\alpha+1)((\epsilon_i/t_i)^{\nu})}\int_0^{W_\theta}\left(\int_0^{L_\theta}c_i^{-\alpha-\nu}dx\right)^{-1}dy\left[(V_{gs} - V_{on})^{\alpha+1} - (V_{gs} - V_{on} - V_{ds})^{\alpha+1}\right]$$

In the presence of contact resistance,

$$I_{d\theta} = \frac{\gamma\mu_0}{(\alpha+1)((\epsilon_i/t_i)^{\nu})}\int_0^{W_\theta}\left(\int_0^{L_\theta}c_i^{-\alpha-\nu}dx\right)^{-1}dy\,\times$$

$$\left[(V_{gs} - V_{on} - I_{d\theta} R_{d\theta})^{\alpha+1} - (V_{gs} - V_{on} - V_{ds} + I_{d\theta} R_{d\theta})^{\alpha+1}\right]$$

Vgs at the onset of conduction for different channel lengths

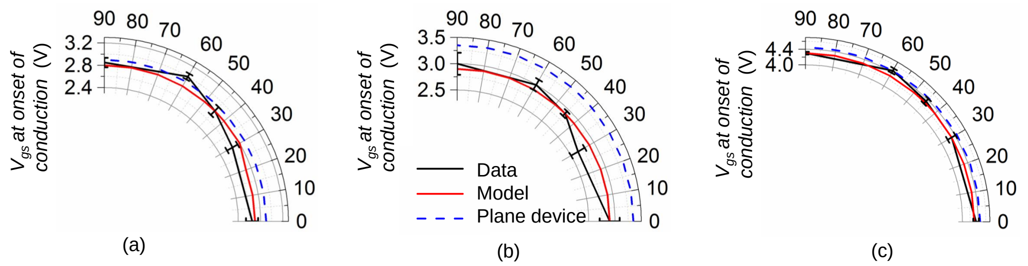

Figure S8: Impact of θ on V_{gs} at the onset of conduction for (a) L=10μm, (b) L=15μm, (c) L=135μm. Parameters extracted from experiment for textured TFTs (black solid line with black markers), elliptical model (red solid line), corresponding parameter measured in planar gate TFT (blue dashed line)
